# ORBMO-RF: a non-destructive classification method for ginseng seeds based on multimodal fusion and improved red-billed blue magpie optimization algorithm

**DOI:** 10.3389/fpls.2025.1743311

**Published:** 2026-01-14

**Authors:** Mingxuan Xue, Yanan Zhu, Bin Liu, Shaozhong Song, Zhongshuai Zhang, Shuqi Zhang, Helong Yu, Liying Wang

**Affiliations:** 1 School of Information Technology, Jilin Agricultural University, Changchun, China; 2 Smart Agriculture Research Institute, Jilin Agricultural University, Changchun, China; 3 School of Data Science and Artificial Intelligence, Jilin Engineering Normal University, Changchun, China; 4 School of Geomatics and Prospecting Engineering, Jilin Jianzhu University, Changchun, China; 5 Institute of Special Animal and Plant Sciences, Chinese Academy of Agricultural Sciences, Changchun, China; 6 National Digital Planting Industry Innovation Sub-Center for Chinese Medicinal Material, Changchun, China

**Keywords:** ginseng seeds, multimodal fusion, non-destructive classification, random forest, red-billed blue magpie optimization algorithm

## Abstract

**Introduction:**

Ginseng, as a precious medicinal plant, requires precise classification of its seeds, which directly impacts production processes and the stability of herbal quality. Furthermore, this classification plays a critical role in advancing ginseng breeding and the modernization of the industry. Current research indicates that systematic automated precision classification technologies for ginseng seeds remain underdeveloped, necessitating breakthroughs in technical bottlenecks.

**Methods:**

This study innovatively proposes a smart classification method based on multimodal data fusion. It employs recursive feature elimination (RFE) to select morphological features from images, followed by competitive adaptive reweighted sampling (CARS) to extract spectral bands from hyperspectral data within the 350~2500 nm range. Morphological and spectral features are then integrated to construct a random forest (RF) classification model optimized using an enhanced, red-billed blue magpie optimization (RBMO) algorithm. To address the RBMO algorithm’s tendency to converge to local optima, the hybrid optimization framework is constructed by integrating three mechanisms: the improved Circle chaotic map, the golden sine search strategy, and the adaptive simulated annealing perturbation mechanism.

**Results:**

Experimental results demonstrate that the proposed model outperforms the baseline model RF, achieving 4.69%、4.79%、4.69 and 4.74% improvements in classification accuracy, precision, recall, and F1-score on test datasets, respectively.

**Discussion:**

The established multimodal data fusion classification system not only provides theoretical and technical foundations for industrial-scale ginseng seed classification but also offers a transferable intelligent decision-making paradigm for non-destructive testing in traditional Chinese medicine.

## 1 Introduction

Seeds are fundamental to agriculture and are often regarded as the “chips” of modern farming. *Panax ginseng C.A. Meyer*, a member of the Araliaceous family, is a highly prized medicinal plant and a renowned tonic herb, commonly referred to as the “King of Herbs” (Gao and Yu, 2021) Ginseng contains ginsenosides and trace elements that contribute to fatigue prevention, antioxidative protection, and cancer therapy, and it plays active roles in the treatment of cardiovascular diseases, immune disorders, and central nervous system dysfunctions (Kang et al., 2009). China is the world’s largest producer of ginseng, accounting for approximately 60–70% of the global annual output (Chen et al., 2022). Ginseng cultivation in China is primarily concentrated in the provinces of Jilin, Liaoning, and Heilongjiang. Due to excessive harvesting and the degradation of suitable habitats, wild ginseng resources have become increasingly scarce, and artificial cultivation has become the main production method. Currently, the seeds primarily used for Panax ginseng cultivation include Cultivated Ginseng Seeds (CGS), Mountain Cultivated Ginseng Seeds (MCGS), and Korean Ginseng Seeds (KGS). In addition to these, American Ginseng Seeds (AGS)—derived from the distinct species Panax quinquefolius—are also prevalent in the market. Although AGS is taxonomically distinct from Panax ginseng, it is frequently encountered in the supply chain, sometimes acting as an adulterant or substitute for true ginseng seeds due to market complexities. These four seed types exhibit highly similar external appearances, making them difficult to distinguish. In particular, the morphological characteristics shared among CGS, KGS, MCGS, and AGS render them virtually indistinguishable to the naked eye. However, these seeds differ significantly in their biological attributes and suitability for specific cultivation methods, and the resulting products vary substantially in quality, yield, market positioning, and economic value. Therefore, the precise and nondestructive classification of ginseng seeds is a critical task in ginseng production, with considerable significance for quality assurance and industrial development. Although research on major crop seeds has been widely reported, studies focusing specifically on ginseng seeds have only begun to emerge in recent years. A recent study (Chen et al., 2025a)showed that hyperspectral imaging combined with transfer learning can effectively distinguish several ginseng seed varieties in a nondestructive manner. This work demonstrates that subtle spectral differences among ginseng seeds can be captured through hyperspectral techniques and modeled using deep networks. However, the method relies solely on hyperspectral data and adopts a two-dimensional spectral–spatial representation, leaving the complementary contributions of RGB morphological features, three-dimensional spatial–spectral context, and multimodal fusion strategies unaddressed. These gaps indicate that the current understanding of ginseng seed characteristics remains incomplete and that further methodological development is needed.

Conventional seed classification methods mainly include morphological and chemical identification approaches. Morphological identification involves classifying seeds based on external traits (Zhang et al., 2007), often relying on the human eye to assess visual characteristics. While this method can provide satisfactory results, it also suffers from several drawbacks, such as inconsistency in classification, slow processing speed, high error rates, and significant labor demands (Sharma et al., 2023). Chemical identification methods include fluorescence scanning techniques (Zilkey et al., 2024), chemical composition analysis, molecular biology techniques such as DNA testing (Nakanishi et al., 2020), and electrophoretic analysis (Montowska and Pospiech, 2007). Among these, fluorescence scanning, chemical profiling, and DNA-based methods offer high classification accuracy (Tang et al., 2022); however, they are generally time-consuming, procedurally complex, and costly. Moreover, they require skilled personnel and sophisticated instruments, making them unsuitable for large-scale, rapid seed identification (Zhang et al., 2023). Given the limitations of these traditional methods in terms of efficiency, cost, and operational complexity, there is an urgent need to develop rapid and nondestructive techniques for ginseng seed classification.

Traditional seed classification methods are often procedurally complex and inefficient. With the advancement of computational technologies, computer vision and spectroscopy have emerged as effective alternatives for seed identification. These technologies have been extensively applied in various domains, including crop breeding, quality evaluation of agricultural products, and the diagnosis of plant diseases and pests (Ambrose et al., 2016; Barreto et al., 2021; Bodor et al., 2023; Cui et al., 2019; Stejskal et al., 2020). The classification results for different technologies are shown in **Table 1**, computer vision-based seed classification typically involves image processing techniques such as binarization, color extraction, and morphological operations to derive external features of seeds, including geometric shape, color, and texture characteristics (Hamid et al., 2022; Li et al., 2025; Tu et al., 2021). For instance, Barrio-Conde et al (Barrio-Conde et al., 2023). developed an image acquisition system and employed deep learning algorithms to classify six varieties of sunflower seeds, achieving an accuracy of 89.5%. In another study, Wang et al (Xiaofei et al., 2024) utilized convolutional neural networks (CNNs) to extract features from rice seed images, followed by classification using a custom-designed model named RiceFastNet, which achieved an accuracy of 96%.

**Table 1:** Classification results of different techniques.

Technical means	Data type	Model	Number of varieties and categories	Accuracy	Ref.
Computer Vision	RGB	DL	six sunflower seed varieties	89.5%	Barrio-Conde (Barrio-Conde et al., 2023)
	RGB	CNN、RiceFastNet	five rice seed varieties	96.0%	Wang (Xiaofei et al., 2024)
Spectral technology	Hyperspectral	SIMCA、KNN、PAC-LA、PCA- QDA	four types of delinted cotton seeds	90.0%	Huang (Huang et al., 2018)
	Hyperspectral	MobileNetV3	four maize seed varieties	93.4%	Zhang (Zhang and Yang, 2023)
	HSI	RBFNN	three maize seed varieties	91.%	Zhao (Zhao et al., 2018)
	VNIR+NIRS	CV-SVM、Elastic Net、(GA)-BP	ten wheat seed varieties	90.4%	Zhang (Zhang et al., 2019)
Computer vision combined with spectral technology	RGB+NIRS-HSI	1D-CNN	Ten types of rice seeds	92.9%	Ye (Ye et al., 2023)
	RGB+NIRS	SVM、RF、KNN	five waxy maize seed varieties	94.0%	Zhuang (Zhuang et al., 2024)

However, external morphological features are often insufficient to capture the intrinsic quality differences among seeds. Spectral techniques, which collect reflectance data across a wide range of wavelengths (Jin et al., 2022), can reveal the internal chemical composition and physical structure of seeds (Ye et al., 2023), providing more and detailed information for seed variety identification (Huang et al., 2018; Li et al., 2020). Compared with traditional image-based approaches, spectroscopy offers advantages in detecting key components such as moisture, protein, and starch content (Bai et al., 2020). Despite these strengths, hyperspectral data are inherently high-dimensional and contain substantial redundant information, which may increase model complexity and computational cost, potentially degrading classification performance. Therefore, selecting informative wavelengths through feature selection and optimization algorithms has become a critical focus in hyperspectral seed classification. Representative studies in other crops have demonstrated the effectiveness of hyperspectral and deep-learning approaches. Zhang et al (Zhang and Yang, 2023) proposed a comparative framework involving four deep learning models—MobileNetV3, VGG16, GoogleNet, and ShuffleNet—for the classification of four maize seed varieties. Among them, MobileNetV3 achieved the highest accuracy of 93.4%. Zhao et al (Zhao et al., 2018). used a hyperspectral imaging system in combination with a radial basis function neural network (RBFNN) to classify three maize seed types, obtaining an accuracy of 91%. Zhang et al (Zhang et al., 2019) developed a classification model based on principal component analysis (PCA) and support vector machines (SVM), achieving an average classification accuracy above 95% for three varieties, approximately 80% for four varieties, and around 66% for six varieties. Chen et al (Chen, 2025b). applied a hybrid 3D-2DCNN model to classify three types of pine seeds, reaching an accuracy of 93.75%.

Single-source data such as individual images or spectral information may overlook critical features. In contrast, multi-feature fusion integrates information from diverse sources, compensating for individual limitations and thereby enhancing the classification capability of models. Consequently, increasing attention has been directed toward multisource data fusion for more accurate and robust seed classification. Li et al (Li et al., 2024). extracted regions of interest from wheat varieties and utilized 3D convolution to capture spatial and spectral features simultaneously, along with 2D convolution for spatial and intrinsic image features. Their method demonstrated superior classification performance, achieving an accuracy of 97.92%. Zhuang et al (Zhuang et al., 2024). combined morphological and spectral features to classify five maize seed varieties using a support vector machine (SVM), reaching an accuracy of 94%. Ye et al (Ye et al., 2023). proposed a hybrid rice seed classification method based on image and spectral information fusion. This image-spectral fusion approach effectively improved classification accuracy of convolutional neural networks (CNNs), achieving 92% accuracy using a one-dimensional CNN (1D-CNN). Due to the advantages of hyperspectral imaging combined with conventional images—such as low cost, nondestructive Ness, ease of operation, and strong adaptability to environmental variations—this technique meets the practical demands for rapid, nondestructive seed classification and enhances grassroots seed management. Therefore, building on existing spectral and image-based approaches, this study adopts a hyperspectral and RGB image data fusion framework to investigate the classification of ginseng seeds, with particular emphasis on feature complementarity and model robustness under optimized parameter configurations.

To achieve optimal performance across various tasks, machine learning models typically require meticulous tuning and proper configuration of their hyperparameters. However, traditional manual tuning methods are often inefficient and heavily dependent on expert experience. Consequently, the integration of intelligent optimization algorithms to automatically search for the optimal hyperparameter combination has become an effective strategy for enhancing model performance. This approach, however, demands not only a deep understanding of the algorithms employed but also the ability to implement appropriate optimization strategies (Yang and Shami, 2020). Li et al (Li et al., 2023). employed a particle swarm optimization (PSO) algorithm to identify optimal parameters for the Gaussian kernel function, which were then applied to a support vector machine (SVM) model for wine classification. The results demonstrated that PSO not only enhanced the training of small-sample datasets but also significantly improved the classification performance of the wine model. Long et al (Long et al., 2022). utilized the whale optimization algorithm (WOA) to optimize an SVM-based fusion model, achieving a notable improvement in the classification accuracy of maize seed vigor. Their approach offers a promising alternative for nondestructive detection of seed aging. Sun et al (Sun et al., 2024). developed a predictive model (ABC-SVM) that integrates the artificial bee colony (ABC) algorithm to assess watermelon seed vigor. The optimized model showed enhanced classification performance and introduced a new strategy for rapid, nondestructive seed vigor detection. These studies collectively demonstrate that swarm intelligence optimization algorithms exhibit strong potential in improving machine learning model performance through effective hyperparameter tuning.

Although substantial research has been conducted on the classification of major crop seeds such as maize, wheat, and rice, and several recent studies have applied hyperspectral imaging and deep or transfer learning techniques to the nondestructive analysis of ginseng-related materials, systematic studies focusing on multimodal RGB–hyperspectral fusion and model optimization strategies for ginseng seed classification remain limited. Chen et al. (Chen et al., 2024) employed a fully connected–convolutional neural network to extract both spectral and spatial cues from hyperspectral images for the nondestructive assessment of Panax ginseng, demonstrating that deep models can effectively characterize subtle spectral differences in ginseng-related materials. Existing studies, both domestic and international, have primarily concentrated on evaluating the quality of ginseng roots, detecting bioactive components, or tracing geographical origin. However, the image and spectral characteristics of ginseng seeds have not yet been thoroughly explored or systematically modeled.

To fill the current research gap in the classification of ginseng seed varieties, this study proposes a novel approach that combines image and hyperspectral features with an enhanced, Red-Billed Blue Magpie Optimization (RBMO) algorithm. The goal is to achieve accurate and nondestructive identification of ginseng seeds. The key areas of focus in this study are as follows:

1. This study fills the gap in the field of ginseng seed classification by pioneering the application of intelligent recognition methods to ginseng seed classification tasks. It establishes a critical bridge between foundational research and industrial applications. The research provides significant theoretical foundations and technical support for enhancing ginseng production efficiency, optimizing seed selection strategies, and strengthening germplasm resource conservation. It holds substantial practical value in advancing the sustainable development of the ginseng industry.
2. The RFE method was applied to select morphological features from images, resulting in the extraction of 10 critical features and achieving a 68.75% reduction in dimensionality. For hyperspectral data, the CARS method was used to extract 13 key spectral bands, reducing dimensionality by 99%. The selected spectral bands exhibited strong chemical correlations with functional groups such as C–H, O–H, and C=C, which correspond to water content, lipids, and carbohydrates in seeds, respectively. These provide a unified spectral basis for classification models with biological interpretability. Image and spectral data demonstrated complementary and correlated feature dimensions. Fusion of these modalities maximizes integration of information from diverse sources, significantly enhancing the completeness of model input features, thereby improving classification discriminative power and stabilizing classification results.
3. A model, ORBMO-RF, was constructed by incorporating a Circle chaotic mapping mechanism, golden sine search strategy,and the adaptive simulated annealing perturbation mechanism. These enhancements optimize the hyperparameters of the Red-Billed Magpie Optimization (RBMO) algorithm for Random Forest (RF) models, significantly improving accuracy. This approach reduces the randomness and subjectivity of manual parameter tuning while enhancing the model’s ability to recognize multimodal data. It provides a novel method for achieving non-destructive detection and precise classification in seed sorting applications.

## 2 Materials and methods

### 2.1 Experimental procedure

**Figure 1:**
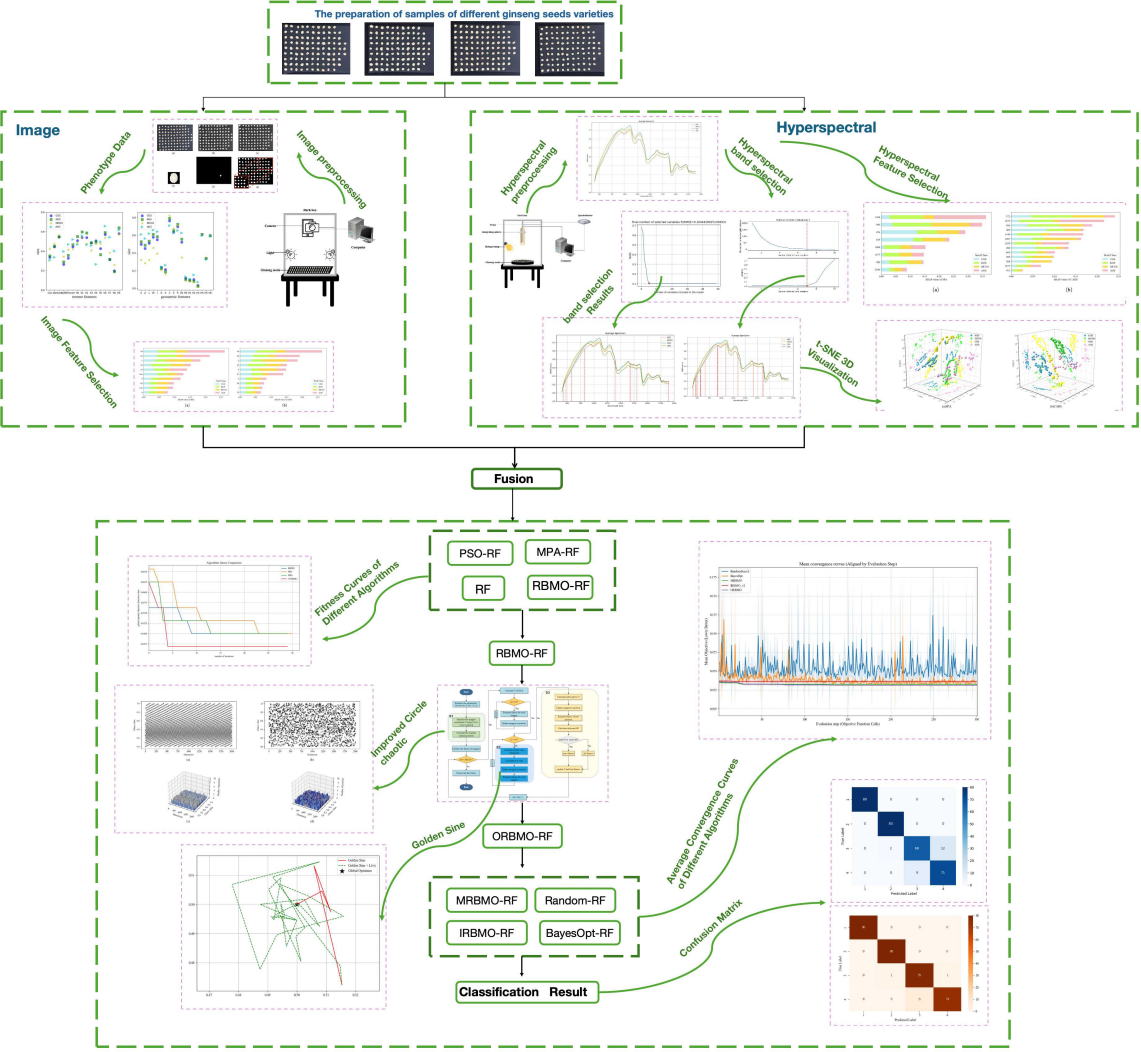
Flowchart of the experimental procedure.

This study was conducted on a 64-bit Windows 10 operating system using Python version 3.12. The computational environment comprised an Intel^®^ Core™ i7-13700KF 3.40 GHz processor, 64 GB of RAM, and an NVIDIA GeForce RTX 4070 Ti SUPER graphics card with 16 GB of VRAM. The overall experimental workflow is illustrated in **Figure 1**.

To achieve effective classification of different ginseng seed varieties, morphological features were first extracted from RGB images. Subsequently, both the extracted morphological features and hyperspectral data were preprocessed, followed by key feature selection from image and spectral information. The morphological and spectral features were then fused and used as inputs for classification models. Different feature fusion strategies were compared to determine the optimal approach. On this basis, a swarm intelligence optimization algorithm was applied to optimize the hyperparameters of the selected machine learning model, thereby improving the model’s overall performance.

### 2.2 Data acquisition

**Figure 2:**
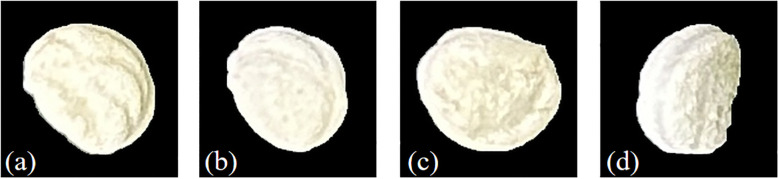
Ginseng seed samples: **(a)**CGS, **(b)** KGS, **(c)** MCGS, **(d)** AGS.

This study focuses on four types of ginseng seeds commonly used in practical ginseng cultivation. The seed samples were obtained from licensed commercial seed suppliers and were taxonomically verified by experts from Jilin Agricultural University to ensure classification accuracy. The four categories include Korean Ginseng Seeds(KGS), Mountain Cultivated Ginseng Seed(MCGS),Cultivated Ginseng Seeds(CGS), and American Ginseng Seeds(AGS), as illustrated in **Figure 2**.

To ensure sample quality, manual screening was conducted during the collection process to eliminate damaged, insect-infested, and impure seeds. Only intact, well-developed seeds of moderate size were retained. A total of 2,000 high-quality seeds were selected for each ginseng variety for use in this study.

#### 2.2.1 Image data acquisition

**Figure 3:**
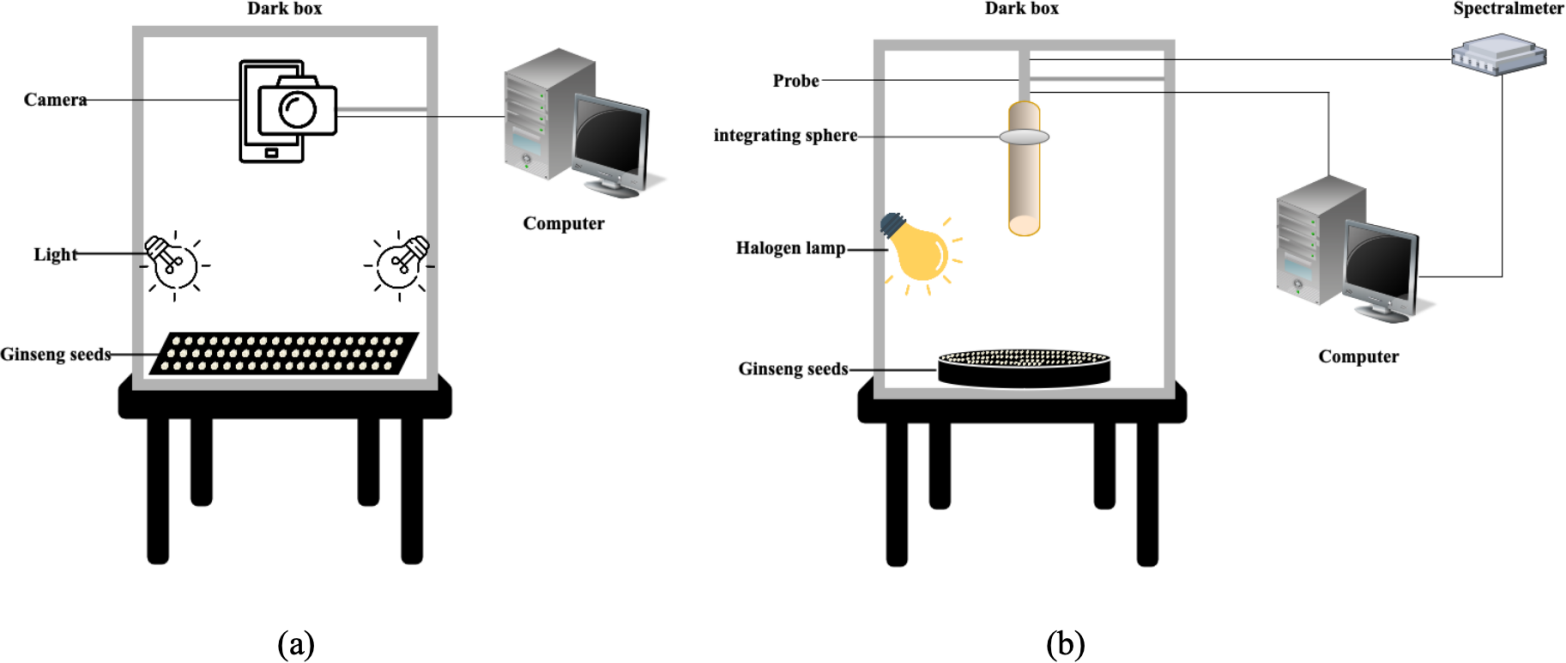
**(a)** Seed image data acquisition schematic diagram **(b)** Schematic diagram of hyperspectral data acquisition for ginseng seeds.

Ginseng seed images were acquired using an iPhone 14 Pro Max with the main rear camera (approximately 48 MP sensor, 24 mm equivalent focal length, f/1.8 aperture). All photographs were taken in the same indoor studio to avoid variation in ambient light. During image acquisition, seeds were placed on a matte black background board, and the smartphone was mounted vertically on a fixed stand with a shooting distance of about 25cm from the lens to the seed surface. Two continuous LED lamps were positioned symmetrically on the left and right sides of the seeds to provide stable and uniform illumination, using neutral white light with color temperature of 4000K.No flash or digital zoom was used. The camera was set to the standard photo mode with an output resolution of 3024 × 4032 pixels (12 megapixels). Autofocus was applied to the seed region, and exposure was controlled automatically under the fixed lighting conditions, with ISO kept at a low level of 200 and shutter speed in the range of roughly 1/125–1/250 s. White balance was set to a fixed daylight/white light mode to keep the color of the seeds consistent across all images. For each ginseng seed type, 100 seeds were randomly oriented and arranged on the background board and photographed as one group, and 20 groups were collected for each variety. The image acquisition setup is shown in **Figure 3a**.

#### 2.2.2 Hyperspectral data acquisition

Hyperspectral data of ginseng seeds were collected using a portable Field Spec 4 spectrometer (ASD Inc., Santa Barbara, CA, USA), covering a spectral range of 350~2500 nm. During measurement, the probe was positioned approximately 10 cm above the seed surface. The instrument’s wavelength accuracy was 0.5 nm, with a repeatability of 0.1 nm. Illumination was provided by a 20 W halogen lamp. The schematic diagram of the data acquisition setup is shown in **Figure 3b** Prior to measurements, spectral calibration was performed using a standard white reference panel. Each spectral data point was obtained by averaging 10 scans with an integration time set at 100 Ms. For each sample group (one plate of seeds), data collection commenced with a scan at the center of the plate. Subsequently, the plate was shifted downward and rotated clockwise in 30°increments for a total of 12 rotations. This protocol yielded 130 spectral acquisitions per group, with two groups collected in total. To maintain consistency, the spectrometer was recalibrated before each measurement, and all operations were performed in a controlled laboratory environment to eliminate ambient light interference.

### 2.3 Data preprocessing

#### 2.3.1 Image data preprocessing

**Figure 4:**
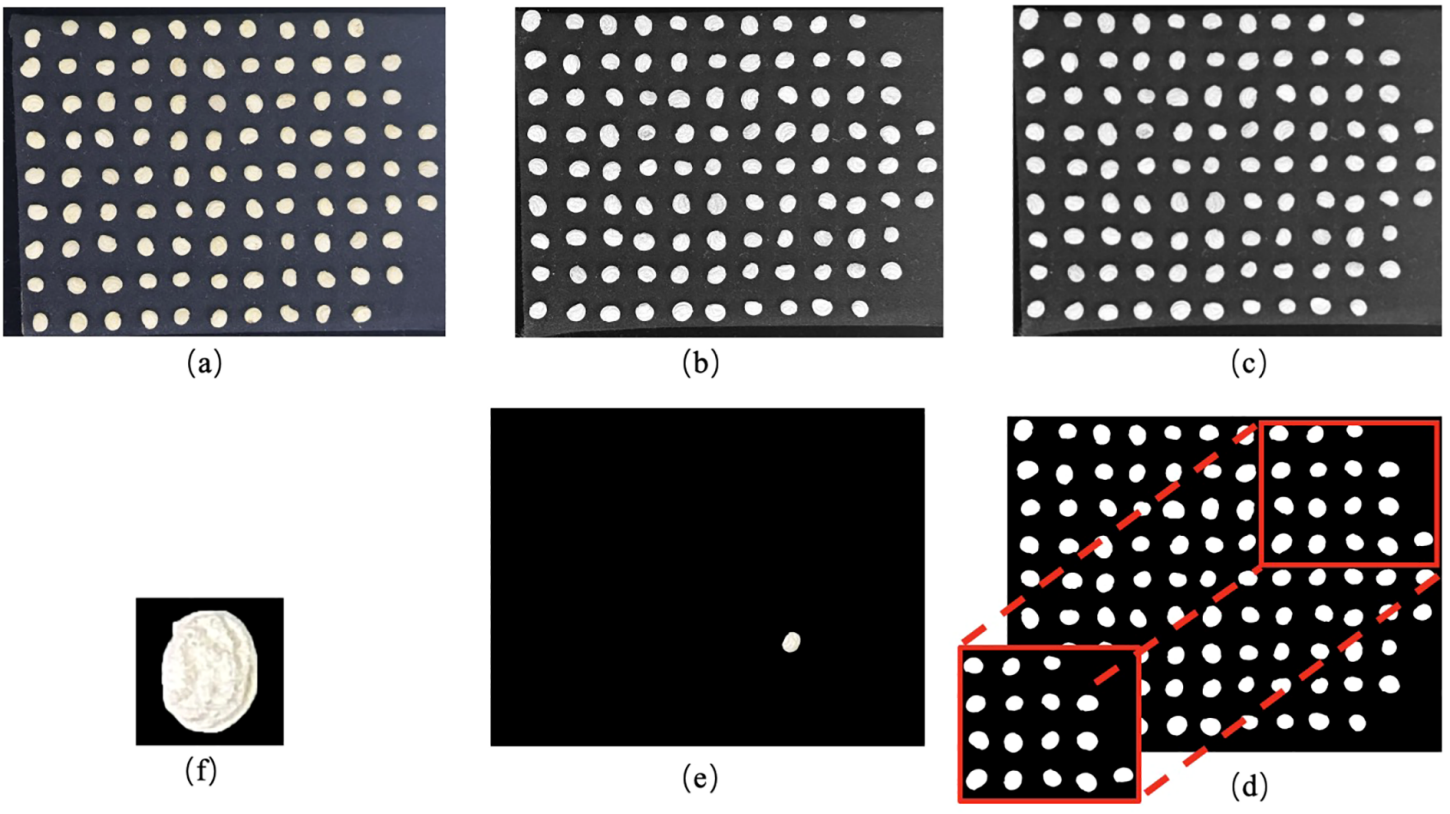
Single ginseng seed extraction process: **(a)** sample photograph; **(b)** grayscale image; **(c)** Gaussian blurred image; **(d)** binary image; **(e)** single seed mask image; **(f)** segmented single seed image.

Previous studies have demonstrated that morphological features play a significant role in seed characterization and are crucial for crop classification. To fully preserve the image information of ginseng seeds, the RGB images were first converted to grayscale, followed by noise reduction using Gaussian filtering. Then, Otsu’s adaptive thresholding method was applied to determine the binarization threshold, generating binary images. Small holes in the binary images were removed through morphological opening operations. Subsequently, the contours of individual seeds were extracted using a boundary tracing algorithm, and their minimum bounding rectangles were calculated. Based on this, each seed contour region was expanded outward by 15 pixels to ensure complete retention of seed image information. Contour-based morphological descriptors (including basic shape- and texture-related measures) were then computed from these regions and used as input variables for the subsequent analysis. The entire image processing workflow is illustrated in **Figure 4**.

#### 2.3.2 Hyperspectral data preprocessing

The surface scattering, light scattering, and other factors of the sample may introduce interference during hyperspectral data acquisition, leading to differences in hyperspectral data between similar samples, which affects the accuracy and stability of classification models. The original hyperspectral data covers the 350~2500 nm spectral range, but due to significant noise and insufficient detector response below 400 nm and above 2400 nm, only the effective 400~2400 nm wavelength range is retained for subsequent analysis to enhance signal quality and model robustness. Therefore, hyperspectral preprocessing is necessary to reduce noise effects, improve data quality, and enhance model classification performance. This study preprocessed the spectral data using Savitzky–Golay smoothing filter (SG smoothing), multivariate scatter correction (MSC), and a combined method of SG smoothing and MSC, which effectively reduced noise interference.

### 2.4 Feature selection

#### 2.4.1 Morphological feature selection

To reduce dataset complexity, enhance the relevance between features and classification outcomes, improve model prediction accuracy, and minimize computation time, this study applied two feature selection methods to the extracted morphological parameters: Recursive Feature Elimination (RFE) and Select from Model (SFM), which selects features based on importance weights.

1. Feature selection based on RFE

RFE was implemented using a supervised learning model as the estimator. In this study, a linear SVM classifier served as the base model, and feature importance was measured by the absolute value of the model coefficients. A 5-fold cross-validation procedure was used to evaluate feature subsets during elimination. At each iteration, the model was refitted and the feature with the lowest importance was removed. The process continued until all features were ranked, and the subset with the highest cross-validation accuracy was selected as the final set.

2. Feature selection based on SFM

SFM was used as a complementary model-based approach. A random forest classifier was employed to compute feature importance scores after model training. Features with importance values below the default threshold in the implementation library were removed, and the remaining features constituted the selected subset. This approach provides an alternative evaluation of feature relevance by exploiting the ensemble structure of tree-based models.

#### 2.4.2 Hyperspectral feature band selection

Hyperspectral data inherently contain many redundant features, and not all spectral bands contribute positively to model classification. Directly using the full feature set for training not only significantly increases computational cost but also adversely affects model performance and generalization. Therefore, selecting the key spectral bands most representative of the classification task is essential to improve both model efficiency and accuracy.

1. Feature extraction based on SPA

The Successive Projections Algorithm (SPA) is an effective feature band selection method widely used in spectral analysis. Its core principle lies in iteratively projecting to maximize the retention of original data information while minimizing the interference caused by redundant features, thereby enhancing the efficiency and accuracy of feature selection. SPA can identify representative wavelength combinations critical for spectral analysis.

2. Feature extraction based on CARS

The Competitive Adaptive Reweighted Sampling (CARS) is a feature wavelength selection method based on the principle of “survival of the fittest.” It integrates Monte Carlo random sampling with partial least squares (PLS) regression. By repeatedly constructing PLS sub-models and calculating their root mean square error of cross-validation (RMSECV), CARS selects the optimal subset of wavelengths corresponding to the minimum error.

### 2.5 Ginseng seed classification model

#### 2.5.1 Random forest classification model

Decision trees form the core components of the RF classification model, which enhances generalization performance by integrating multiple decision trees. During tree construction, the model randomly selects a subset of features at each node to determine the optimal split. Each decision tree independently classifies the input sample, and the final output is determined by aggregating the results of all trees through majority voting or averaging. Random Forests are particularly effective in handling high-dimensional data and large-scale training samples, significantly reducing the risk of overfitting.

In practical applications, model hyperparameters are often selected manually, which can introduce subjectivity and randomness. To improve the accuracy of hyperparameter selection and reduce human intervention, this study employs a swarm intelligence optimization algorithm to optimize the Random Forest model. By iteratively searching for the optimal parameter set, the algorithm enhances classification accuracy. This approach not only improves the precision and efficiency of hyperparameter tuning but also ultimately leads to superior classification performance.

#### 2.5.2 Red-billed blue magpie optimization algorithm and its improvements

The Red-Billed Blue Magpie Optimization (Red-billed Blue Magpie Optimization, RBMO) algorithm is a swarm intelligence optimization method inspired by the collective foraging behavior of red-billed blue magpies. This algorithm emulates the species’ natural behaviors during predation, including searching, chasing, attacking, and food storing. The optimization process consists of four main stages: population initialization, group foraging, cooperative hunting, and food storage.

The RBMO algorithm offers notable advantages, including efficient global search capabilities and a dynamic competition–cooperation mechanism. However, in the later stages of iteration, the magpie population is prone to inertia-driven search behavior, which may lead to uneven population distribution and premature convergence to local optima. Moreover, in complex classification tasks, a mismatch may arise between the search direction and the topological structure of the feature space.

In the population initialization phase of the RBMO algorithm, individual positions are typically distributed randomly, which may result in an uneven distribution across the search space. To enhance the initial diversity of the population, this study incorporates the Circle chaotic mapping mechanism. This mapping ensures a uniform distribution of individuals within the constrained search space, offering strong ergodicity and improved coverage. As a result, it significantly enhances the algorithm’s global exploration ability in the early stages and increases the likelihood of identifying the global optimum. The mathematical formulation is Equation 1:

(1) $$\begin{array}{c} x_{i+1}=mod(x_{i}+b-(\frac{a}{2\pi }\cdot \sin2\pi x_{i}),1),x_{i}\in [0,1] \end{array}$$

Where the parameters a=0.5, b=0.2, the mapping generates sequences with chaotic properties in the interval [0,1], which are used to initialize the positions of population individuals.

**Figure 5:**
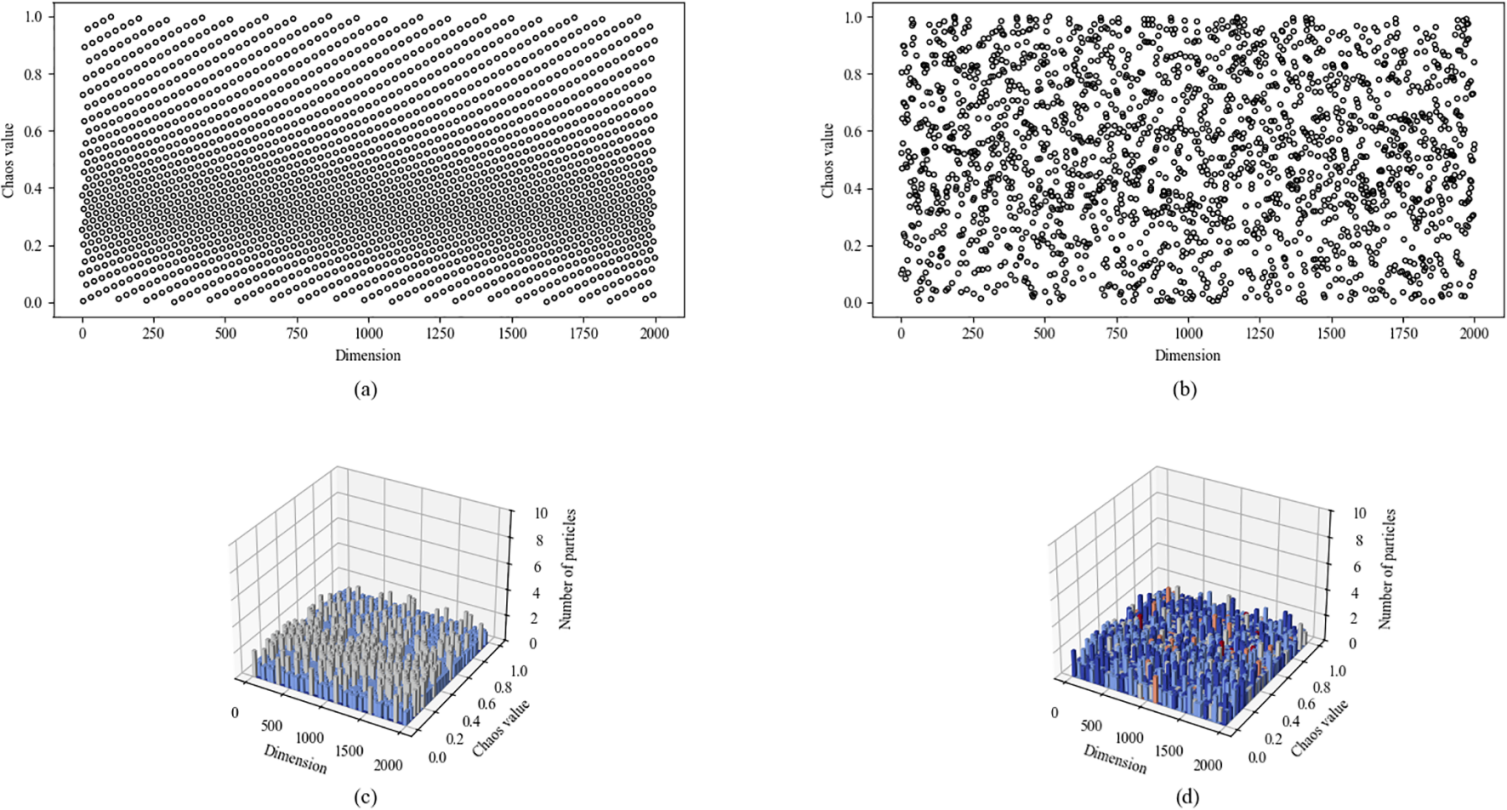
Scatterplot and histogram of frequency distribution for improved Circle chaotic mapping. **(a)** Circle chaotic map scatter plot; **(b)** Improved Circle chaotic map scatter plot; **(c)** Frequency histogram of Circle chaotic map; **(d)** Frequency histogram of Improved Circle chaotic map.

From subfigures (a) and (c) of **Figure 5**, it can be observed that the initial particles generated by the Circle chaotic map are primarily concentrated in the interval [0.2, 0.5]. However, an overly concentrated distribution of initial candidate solutions would significantly reduce the population diversity of the RBMO algorithm. To address this issue, this paper improves the original Circle chaotic map mechanism, with its mathematical expression as shown in Equation 2:

(2) $$\begin{array}{c} x_{i+1}=mod(2.75x_{i}+b-(\frac{a}{2.75\pi }\cdot sin(2.75\pi x_{i}),1)),x_{i}\in [0,1] \end{array}$$

The parameters are set as a=0.6 and b=0.3, and the mapping generates a sequence with chaotic characteristics within the interval [0,1], which is used to initialize the positions of individuals in the population.

Subplots (b) and (d) in **Figure 5** illustrate the initial candidate solution distribution and the corresponding frequency histogram generated by the improved Circle chaotic mapping operator. Compared with the standard Circle chaotic map, the improved mapping produces a more evenly dispersed particle distribution, resulting in a broader coverage of the search space. This distributional characteristic indicates that the improved Circle chaotic mapping can provide a more diverse initial population for RBMO, which is expected to benefit the global exploration phase of the optimization process.

In the prey attack phase of the RBMO algorithm, the exploration capability of the search path plays a critical role in solution accuracy. To enhance local search precision while maintaining global search ability, this study introduces the Golden Sine Search (GSS) strategy. This approach integrates the golden ratio coefficient with a sine-based perturbation mechanism, ensuring precise search direction while introducing nonlinear disturbances to increase diversity. The position update formula is Equation 3:

(3) $$\begin{array}{c} x^{i}(t+1)=x^{i}(t)+r\cdot \sin(\theta )\cdot (\varphi \cdot x^{food}(t)-x^{i}(t)) \end{array}$$

Where: $\varphi =\frac{\sqrt{5}-1}{2}$ is the golden ratio constant; r∈[0,1] is a uniformly distributed random number; θ∈[0,2π] is a sinusoidal perturbation angle; and $x^{food}(t)$ denotes the position of the current optimal individual. The strategy guides individuals to oscillate periodically in the convergence direction to avoid monotonous search paths, thus improving the exploitation of the target region and convergence stability.

**Figure 6:**
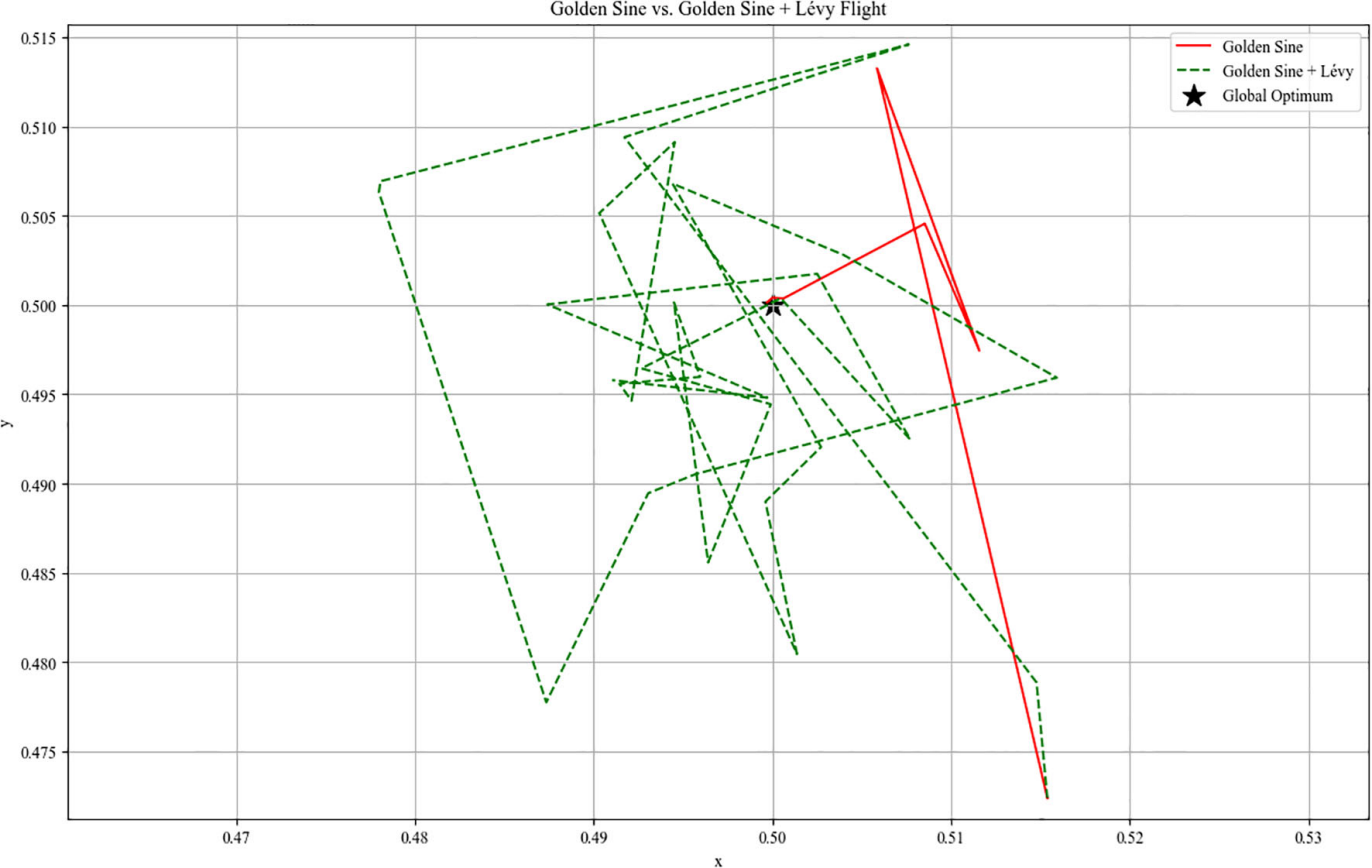
Schematic diagram comparing the search trajectory with Lévy Flight strategy against the golden sine-based path.

Despite the inherent leaping capability of the Golden Sine search strategy, it tends to become trapped in repetitive searches within local regions when approaching the optimal solution. As shown in **Figure 6**, the red line segments illustrating the Golden Sine search trajectory indicate that it exhibits issues of short path length and restricted search scope after approaching the target area.

To enhance the ability to escape boundary regions and local extremum traps, this study introduces a Lévy Flight perturbation mechanism and constructs the following hybrid update strategy, as shown in Equation 4:

(4) $$\begin{array}{c} x^{i}(t+1)=x^{i}(t)+r\cdot \sin(\theta )\cdot (\varphi \cdot x^{food}(t)-x^{i}(t))+\alpha \cdot Lévy(\lambda ) \end{array}$$

The Lévy(λ) distribution represents the Lévy distribution step length, where α denotes the perturbation strength. The Lévy distribution exhibits long-tail characteristics, enabling intermittent large-step jumps.

**Figure 6** illustrates the search trajectories of GS (red solid line) and the improved GS-LF (green dashed line) within a two-dimensional objective function space. It can be observed that while GS converges rapidly to a near-optimal region, its path exhibits a tendency toward systematic contraction overall. In contrast, the GS-LF strategy demonstrates stronger spatial exploration capabilities, with its trajectory maintaining jumps even in proximity to the optimal solution, thereby enabling fine-grained search capabilities. From an algorithmic performance perspective, the Lévy mechanism does not disrupt the original convergence trend of the golden sine algorithm but instead enhances boundary exploration and local refinement capabilities in later stages by controlling jump probabilities or perturbation amplitudes. Consequently, integrating the GS-LF strategy into the golden sine phase of the RBMO Algorithm exhibits excellent algorithmic compatibility and synergistic potential, anticipated to enhance overall optimization precision and stability.

To enhance the RBMO algorithm’s ability to escape local optima during the later stages of the search process, this study introduces the Simulated Annealing (SA) strategy as a perturbation mechanism. Inspired by the Metropolis criterion in physical annealing, this mechanism allows the acceptance of inferior solutions with a certain probability when the new fitness value after an individual update is worse than the previous one, thereby preserving diversity in the search space. The acceptance probability function is defined as Equation 5:

(5) $$\begin{array}{c} P=exp(-\frac{\Delta f}{T}) \end{array}$$

Where: $\Delta f=f(X^{new})-f(X^{old})$ denotes the difference between the old and new solution fitness; T is the current temperature, which satisfies the linear annealing process:

(6) $$\begin{array}{c} T=T_{0}\cdot (1-\frac{t}{T_{max}}) \end{array}$$

In Equation 6: $T_{0}$=100 is the initial temperature, t is the current iteration number, and $T_{max}$ is the maximum iteration number. The simulated annealing mechanism enables the algorithm to effectively jump out of the local optimal trap in the convergence stage, improve the diversity of the final solution and the global optimal probability, and thus improve the overall optimization performance.

However, directly integrating traditional SA into the RBMO framework poses an issue: the fixed temperature decay strategy struggles to adapt to the varying search rhythms across different phases. To address this, this study adopts Adaptive Simulated Annealing (ASA) as a replacement for classical SA. ASA introduces a dynamic adjustment mechanism in temperature updates, enabling the annealing temperature’s decay rate to automatically adapt based on the current search state. Specifically, ASA dynamically adjusts the annealing temperature by incorporating the gap between the current individual’s fitness and the global optimal solution, as shown in Equation 7:

(7) $$\begin{array}{c} T=T_{0}\cdot \exp(-\beta t) \end{array}$$

In this context, β controls the cooling rate to prevent premature convergence.

In summary, ASA retains more feasible solution paths in the global exploration phase and avoids premature freezing during local convergence compared to SA, resulting in a more stable and robust overall optimization process. Particularly in image classification scenarios, the ASA mechanism effectively enhances the diversity of solutions in high-dimensional parameter spaces, significantly improving the generalization capability of classifiers. When combined with RBMO, the ASA strategy complements the blue jay behavior model, effectively addressing issues such as early convergence and insufficient jump amplitude, validating its fusion potential and performance enhancement value in complex classification tasks.

**Figure 7:**
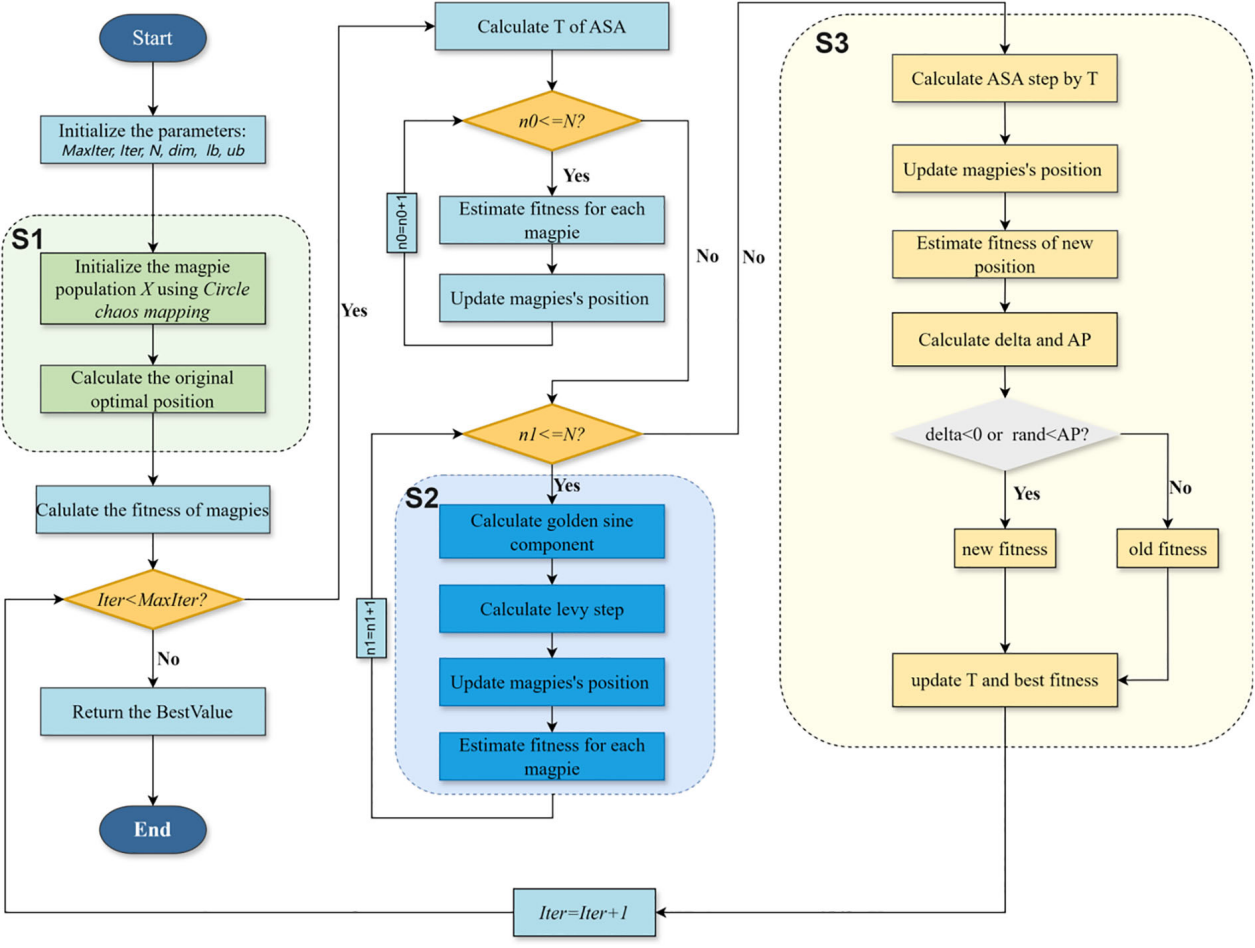
Flowchart of the ORBMO algorithm.

The three mechanisms optimize the RBMO algorithm’s search capability and stability at different stages: the Circle chaotic mapping enhances the quality of the initial population distribution; the Golden Sine Search improves local exploitation and search path diversity; and the Simulated Annealing mechanism effectively increases the algorithm’s ability to escape local optima. Collectively, these enhancements significantly improve the RBMO algorithm’s global optimization capability and convergence accuracy. Under the overall influence, the global optimization capability and convergence accuracy of the RBMO algorithm are significantly enhanced, with the specific flowchart shown in **Figure 7**.

#### 2.5.3 ORBMO-RF based classification model

RF demonstrates excellent performance in classification tasks owing to its ensemble learning mechanism, which incorporates random sampling and majority voting strategies. Prior studies have indicated that the classification accuracy of the model largely depends on two critical hyperparameters: the number of learners (i.e., the number of decision trees) and the sampling dimension of the feature space (i.e., the maximum proportion of features available to each tree). However, optimal settings for these hyperparameters typically vary according to specific application scenarios. An insufficient number of trees may reduce model diversity, limiting the ability to capture complex data distributions effectively; conversely, an excessive number increases computational cost and reduces efficiency. Similarly, the choice of feature subset size requires balancing information retention and feature redundancy: too low a sampling rate risks losing key information, while too high may decrease diversity among sub models, leading to increased bias and compromised classification performance.

To address these challenges, this study innovatively employs the RBMO algorithm for adaptive hyperparameter tuning. The ORBMO-RF includes the following core steps:

1. Initialize the population with a population size of n=30, set the maximum number of iterations T = 100, define the problem dimension dim=3, and specify the search interval lower bounds lb=[10,3,2] and upper bounds ub=[300,30,20].
2. Employ an innovative Circle chaos mapping strategy to initialize population positions by generating chaotic sequences within [0,1], which are then mapped to the corresponding intervals of each dimension to ensure uniform coverage and initial diversity of the parameter space. Calculate the fitness of each individual and record the current optimal solution.
3. Based on the hierarchical collaboration mechanism of the Red-billed Leiothrix, divide the population into “leaders” and “followers” and dynamically adjust the search direction using position update formulas to simulate the coordinated foraging behavior of leaders and followers in the population.
4. To prevent premature convergence, introduce an adaptive switching probability combined with a Lévy flight step size and golden sine search strategy to perform fine-tuned exploitation of the neighborhood of the current optimal solution, thereby enhancing local search capability and convergence precision.
5. Enforce boundary constraints on the updated individual positions to ensure all hyperparameters remain within the predefined search range and eliminate invalid solutions.
6. Apply adaptive simulated annealing perturbations to the current global optimal solution by dynamically superimposing Gaussian disturbances controlled by temperature adjustments to accept worse solutions and escape local optima, thereby strengthening late-stage fine search and global optimization capabilities.
7. If the fitness of the perturbed new solution surpasses the original global optimum, update it; otherwise, retain the original solution to prevent excessive disruption of existing optimal solutions and maintain convergence stability.
8. Implement a “food storage” mechanism: record the optimal hyperparameter combinations and their fitness values obtained during the current iteration and update the historical optimal solution set.
9. Repeatedly execute steps 3~8 until the maximum number of iterations is reached, or no significant fitness improvement occurs over consecutive generations, triggering an early termination criterion.
10. Terminate the optimization process, output the optimal RF hyperparameters, construct a high-performance random forest classification model, and enhance classification accuracy and generalization ability.

### 2.6 Evaluation metrics for classification models

The evaluation metrics used in this study are calculated based on the data derived from the confusion matrix and serve to assess the classification performance of the models. These metrics include Accuracy (Acc), Precision (P), Recall (R), and F1-score (F1), as shown in Equations 8–11.

1. Classification Accuracy

Accuracy represents the proportion of correctly predicted samples out of the total number of samples, reflecting the overall correctness of the classification model. It is calculated as follows:

(8) $$\begin{array}{c} Acc=\frac{TP+TN}{TP+FP+FN+TN} \end{array}$$

Where TP is the number of samples correctly predicted as positive samples; TN is the number of samples correctly predicted as negative samples; FP is the number of samples incorrectly predicted as positive samples; and FN is the number of samples incorrectly predicted as negative samples.

2. Precision

Precision represents the proportion of correctly identified positive samples among all samples predicted as positive by the model. It is calculated as follows:

(9) $$\begin{array}{c} P=\frac{TP}{TP+FP} \end{array}$$

Where TP is the number of samples correctly predicted as positive samples; FP is the number of samples incorrectly predicted as positive samples.

3. Recall

Recall represents the proportion of true positive samples correctly identified by the model among all actual positive samples. It is calculated as follows:

(10) $$\begin{array}{c} R=\frac{TP}{TP+FN} \end{array}$$

Where TP is the number of samples correctly predicted as positive samples; FN is the number of samples incorrectly predicted as negative samples.

4. F1-score

The F1-score is the harmonic mean of precision and recall, used to comprehensively evaluate the accuracy and completeness of a model. *It* is calculated as follows:

(11) $$\begin{array}{c} F1=\frac{2\cdot P\cdot R}{P+R} \end{array}$$

## 3 Results

### 3.1 Data preprocessing results

#### 3.1.1 Image data preprocessing results

Image processing techniques were employed to extract geometric and texture features from 2000 individual ginseng seeds per variety to characterize their morphological attributes, where the contour-based feature extraction follows conventional methods. To match the spectral data acquisition, 260 seeds were randomly and non-repeatedly selected from these 2000 seeds for subsequent joint analysis, and were further divided into 180 samples for training and 80 samples for testing. Geometric features, which reflect the genetic and biological properties of seeds, included 18 key parameters: perimeter (S), area (A), major axis length (L), minor axis length (W), inscribed Circle radius (r), aspect ratio (K), dispersion (e), circularity (C), elongation (E), rectangularity (R), equivalent Circle diameter (Ed), and Hu invariant moments(including 7 parameters,H0~H6). Texture features, describing the overall surface structure of the image, comprised another set of 16 key parameters including contrast(Con), dissimilarity(Dis), homogeneity(Hom), angular second moment (ASM), energy(Ene), correlation(Corr), and local binary pattern (LBP, including 10 parameters,hist0~hist9), comprehensively capturing the texture information of ginseng seeds.

**Figure 8:**
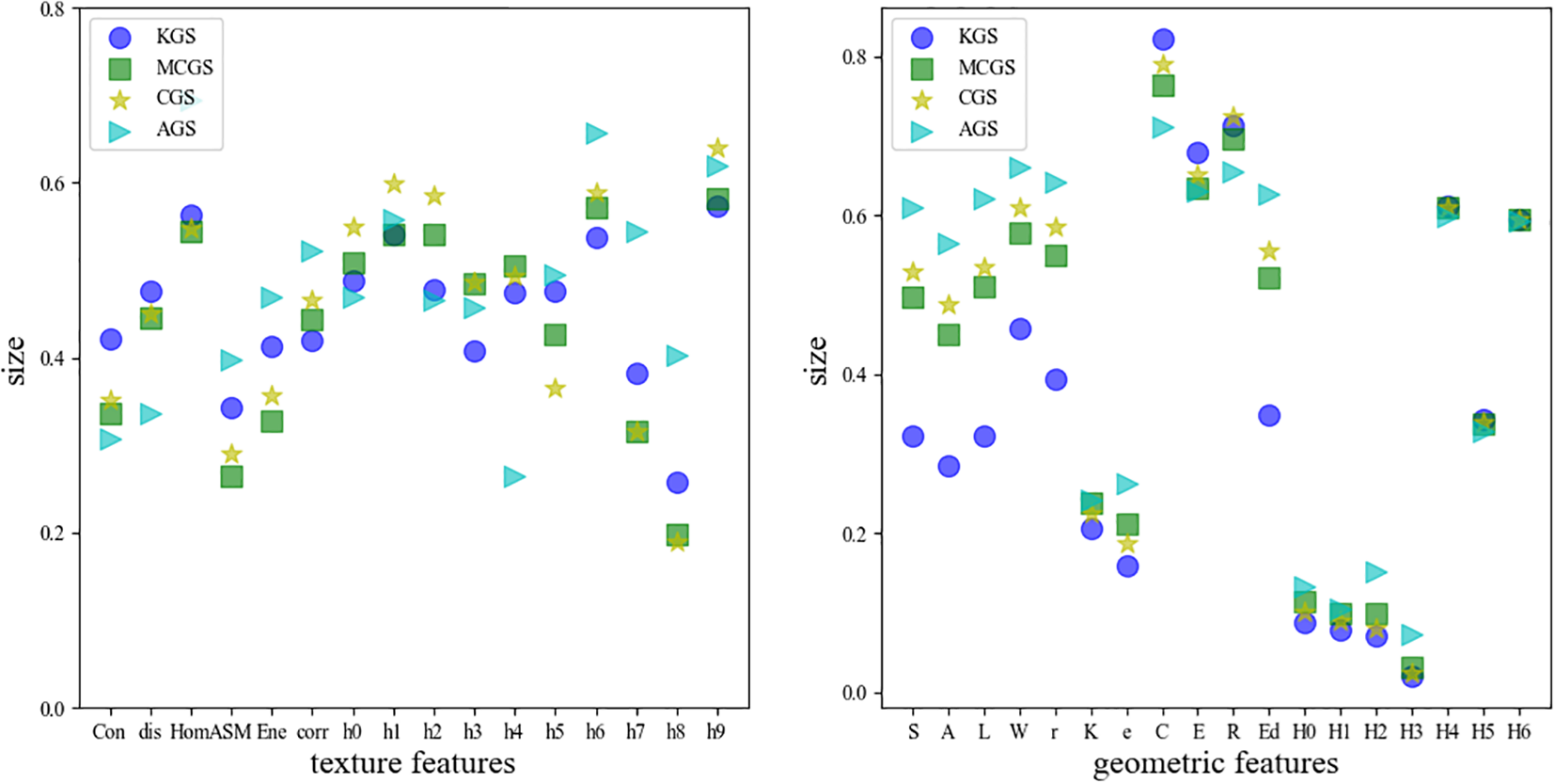
Mean morphological features of different seed categories: **(a)** mean texture features; **(b)** mean geometric features.

A total of 34 morphological features were extracted from ginseng seeds. **Figure 8** illustrates the normalized mean values of these features across different seed categories. It is evident that most ginseng seed varieties exhibit significant differences in their morphological feature distributions.

**Figure 8a** presents the average texture features of different seed types. American ginseng seeds exhibit lower contrast and dissimilarity values, while KGS show higher average values for hist0, hist1, and hist2. **Figure 8b** displays the average geometric features for the different seed types. Compared to other categories, under forest ginseng seeds have notably smaller geometric features, whereas AGS possess larger ones. In summary, distinguishing ginseng seed varieties based on morphological features is feasible.

Based on the classification performance of morphological features presented in **Table 2**, this study conducted a systematic evaluation of the 34 extracted features across three classification models using cross-validation experiments. The results demonstrated that the RF model outperformed both Support Vector Machine (SVM) and K-Nearest Neighbors (KNN) in key metrics, achieving a classification accuracy of 52.50% and a precision of 54.46%. Accordingly, the RF algorithm was selected as the baseline framework for seed classification. In subsequent stages, swarm intelligence optimization algorithms were integrated to adaptively fine-tune the model’s hyperparameters, aiming to further enhance its generalization performance.

**Table 2:** Comparison of different classification models.

Model	Result			
	A	P	R	F1
RF	0.5250	0.5446	0.5250	0.5316
SVM	0.5000	0.6177	0.5000	0.4988
KNN	0.4917	0.5591	0.4917	0.4996

#### 3.1.2 Hyperspectral data preprocessing results

The study conducted spectral preprocessing using three methods: Savitzky–Golay smoothing filter (SG smoothing), multivariate scatter correction (MSC), and a combination of SG smoothing and MSC. These methods effectively reduce noise interference. As shown in **Table 3**, among the three classification models, the RF model combined with SG preprocessing achieved the highest classification accuracy of 89.67%, outperforming both MSC and SG+MSC. Therefore, RF was selected as the baseline classifier for subsequent modeling.

**Table 3:** Comparison of preprocessing methods across different models.

Model	SG	MSC	SG+MSC
RF	0.8967	0.8583	0.8683
SVM	0.7589	0.8487	0.8567
KNN	0.8423	0.8347	0.8207

**Figure 9:**
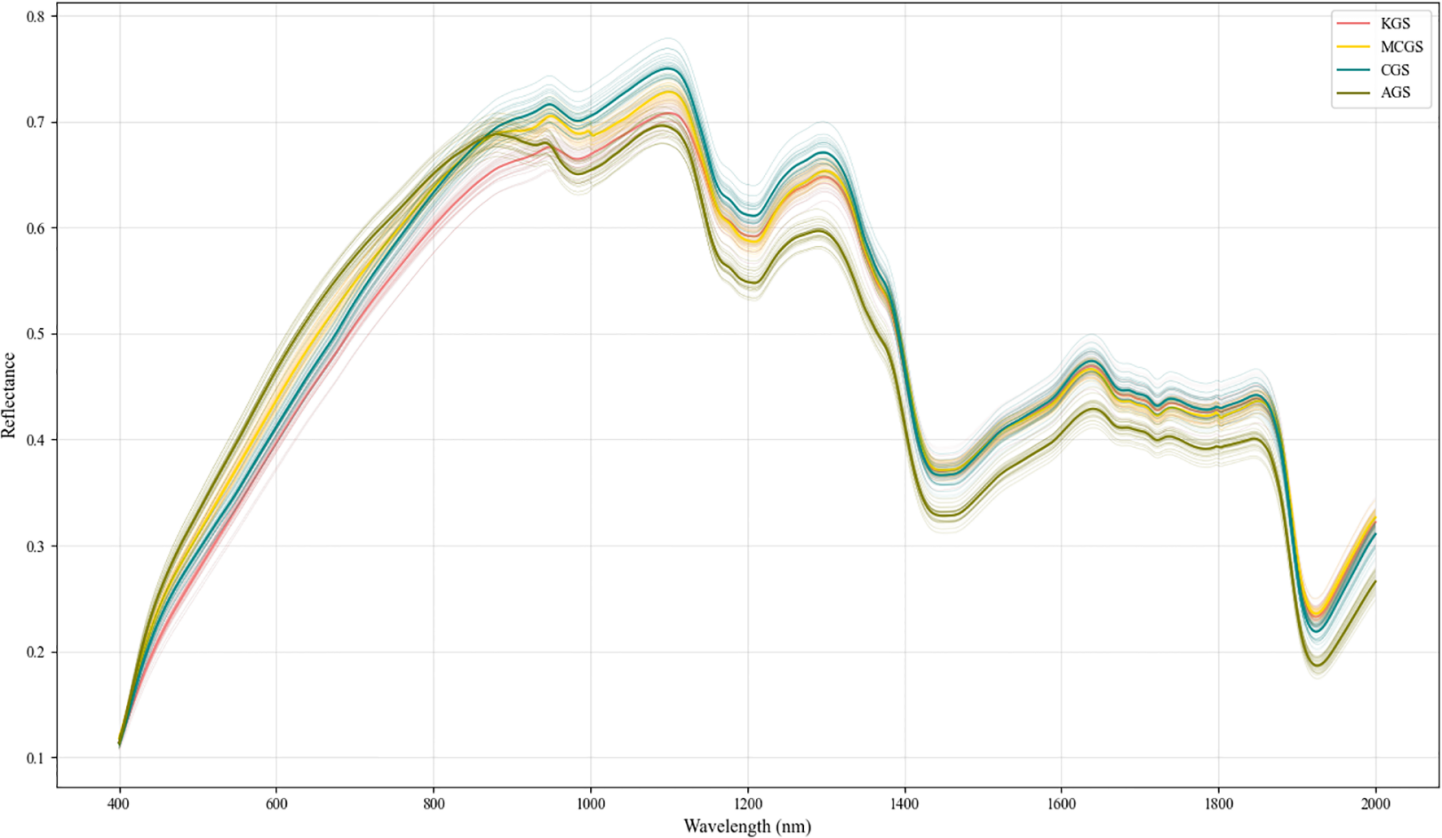
Preprocessed spectra.

The SG-smoothed spectral curves are shown in **Figure 9**. The overall trends of the SG-smoothed spectral curves are consistent, but subtle differences exist in the key absorption bands (1000~1300 nm, 1400~1900 nm, 2000~2300 nm). These differences reflect variations in the chemical composition of ginseng seeds, providing crucial evidence for subsequent spectral feature extraction and substance identification.

### 3.2 Classification based on morphological features of ginseng seeds

#### 3.2.1 Extraction and analysis of morphological feature parameters

**Figure 10:**
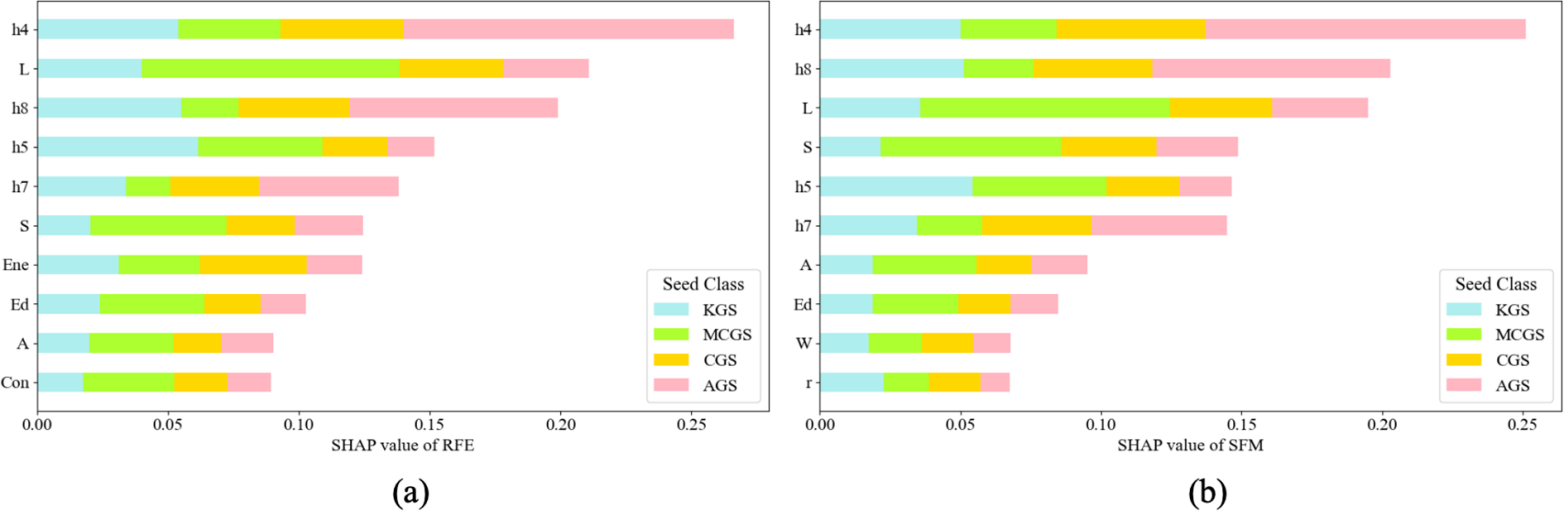
SHAP contributions of morphological feature selection for different ginseng seed varieties:**(a)** RFE **(b)** SFM.

The most important features selected from 32 morphological features using RFE are shown in **Figure 10a**, including: hist4, hist8, hist5, hist7, ASM, Contrast, Ed, L, A, and S. Among these, AGS consistently exhibited the highest SHAP values in hist4 and hist8, indicating significant morphological differences likely attributable to their fuller seed surfaces and distinct length ratios. KGS and CGS showed relatively similar SHAP distributions for these traits, suggesting greater morphological similarity.

The most important features selected from 32 morphological features using SFM are shown in **Figure 10b**, including: Ed, L, A, r, W, S, Hist4, Hist8, Hist7, and Hist5. Among these, AGS maintained the highest contribution for traits hist4, hist7, and hist8, demonstrating significant morphological separation characteristics. Although W and Ed exhibited minor contributions in the model, they were retained, indicating their auxiliary role in distinguishing KGS from MCGS.

Overall, hist8, hist4, L, and hist5 ranked highly in both the RFE and SFM models, indicating these are key features for distinguishing ginseng seed types. Among them, AGS and MCGS exhibited the most pronounced differences in these characteristics. SHAP analysis revealed the interpretability of model decisions, providing scientific support for the quantitative morphological classification of ginseng seeds.

#### 3.2.2 Morphological feature-based classification of ginseng seeds using random forest

All normalized morphological features of ginseng seeds, along with the selected features, were input into the RF model. The dataset was split into training and testing sets at a ratio of 7:3 for subsequent analysis. For the RF model, the range of n_estimators were set to 1~50, while the range of max_features was set to 1~100. After a limited number of iterations, the optimal fitness value was obtained, and consequently the optimal values of n_estimators and max_features for the classification model were determined (the subsequent partitioning followed this ratio). The performance of the classification model is presented in **Table 4**.

**Table 4:** Classification results of random forest using morphological features.

Selection algorithm	Result			
	A	P	R	F1
None	0.5385	0.5612	0.5385	0.5455
RFE	0.5737	0.6026	0.5737	0.5839
SFM	0.5001	0.5328	0.5001	0.5120

As shown in **Table 4**, RFE demonstrated superior performance in feature selection compared with SFM. When only ten key features were retained through RFE, the classification performance improved relative to using the full set of morphological features. This result indicates that appropriate feature selection can effectively enhance the classification performance of the model. However, due to the high visual similarity of ginseng seeds, certain categories exhibit ambiguous boundaries, which increases the difficulty of discrimination and leads to partial misclassification. This also highlights the limitations of relying solely on morphological features for classification, particularly when the number of categories increases. Therefore, to further improve classification accuracy, it is necessary to incorporate more comprehensive information sources, such as spectral data, for integrated analysis.

### 3.3 Classification of ginseng seeds based on hyperspectral data

#### 3.3.1 Spectral feature band selection and analysis

**Figure 11:**
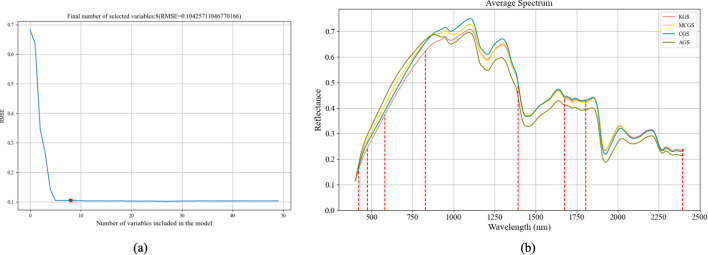
Feature wavelength selection for ginseng seed classification using the SPA algorithm. **(a)** RMSE curve showing the variation in error with the number of selected wavelengths.**(b)** Index positions of the eight selected characteristic wavelengths.

The Successive Projections Algorithm (SPA) was used to screen representative wavelengths from the 2,000 spectral bands ranging from 400 nm to 2,400 nm. The wavelength selection process is illustrated in **Figure 11**. **Figure 11a** shows that the root mean square error (RMSE) reaches its minimum when selecting eight feature bands, with the lowest point marked by a red circle. **Figure 11b** presents the precise positions of the selected feature bands within the spectral index.

**Figure 12:**
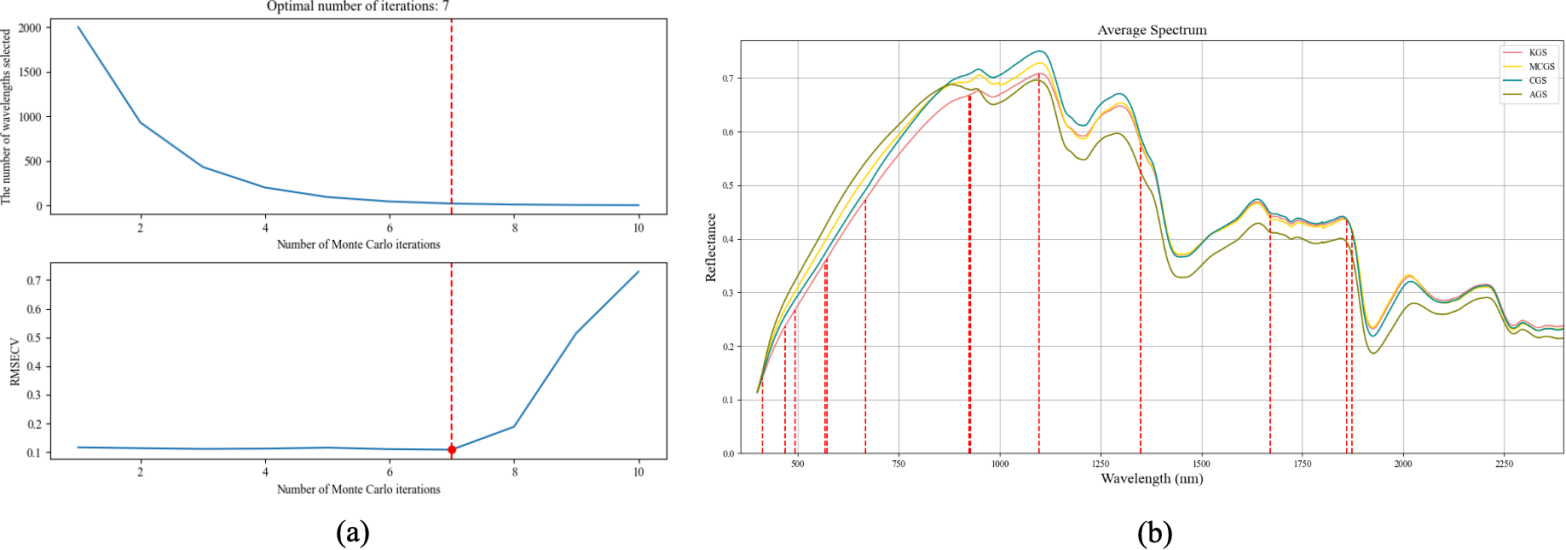
Feature wavelength selection for ginseng seed classification using the CARS algorithm. **(a)** Variation curve of RMSECV with the number of selected wavelengths. **(b)** Index positions of the 13 selected characteristic wavelengths.

Using the CARS algorithm, representative spectral bands were selected from the 2,000 wavelengths ranging from 400 nm to 2,400 nm. The wavelength selection process is illustrated in **Figure 12**. As shown in **Figure 12a**, the number of selected wavelengths decreases progressively with the number of iterations. The optimal subset is obtained at the 7th iteration, highlighted by a red box, effectively eliminating redundant wavelengths irrelevant to seed classification. **Figure 12b** illustrates the spectral index positions of the final 13 selected wavelengths.

Furthermore, **Figures 11** and **12** illustrate the sets of characteristic wavelengths selected by the SPA and CARS methods, respectively, for classifying different Panax ginseng seed varieties. The SPA method reduced the original 2,000 spectral bands to just 10, effectively achieving a dimensionality reduction of approximately 99.6%. In comparison, the CARS method identified 13 key wavelengths, reducing the number of input features by around 99%. Both approaches substantially decreased the dimensionality of the input features, thereby enhancing modeling efficiency and computational performance.

**Figure 13:**
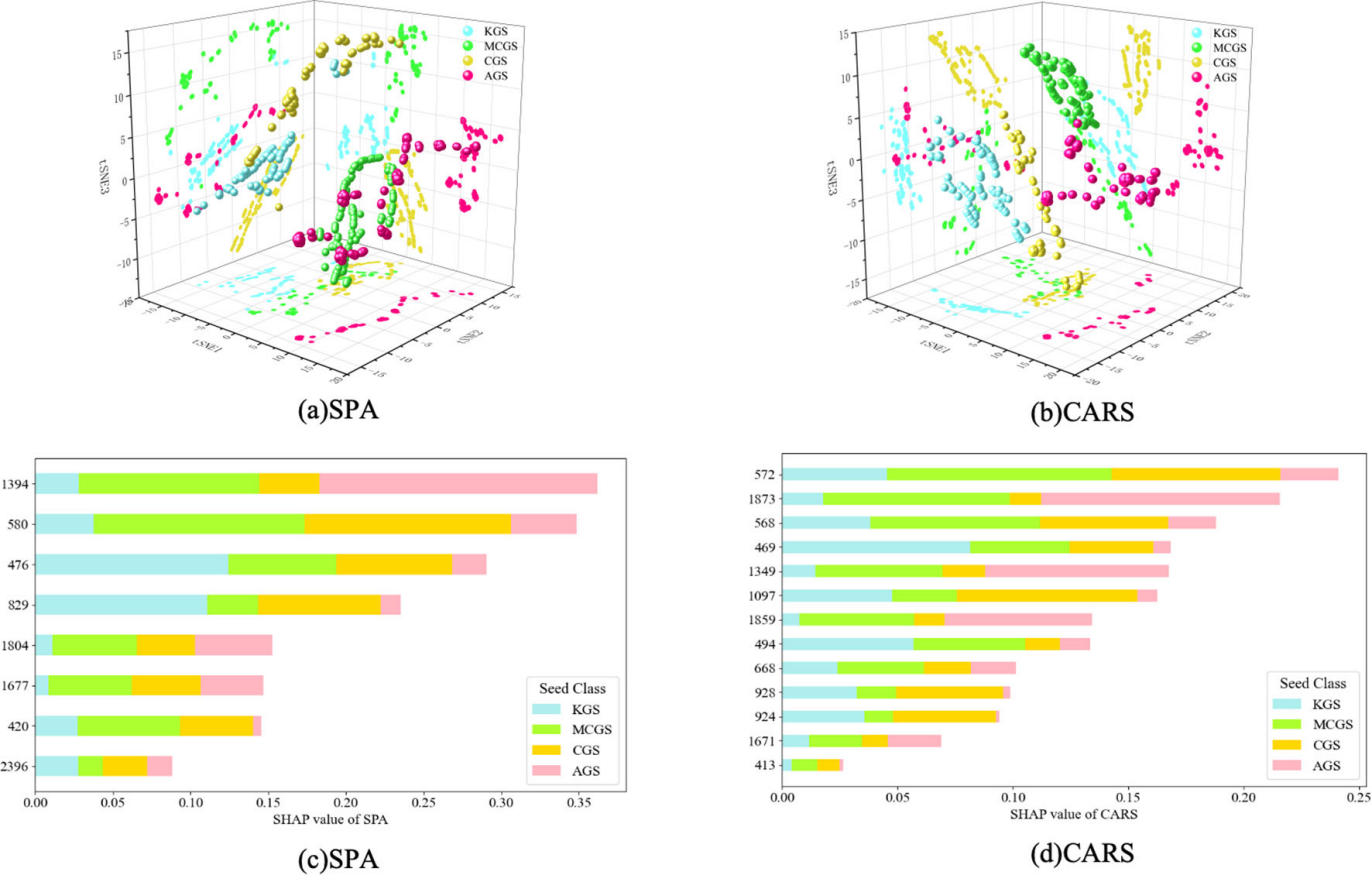
t-SNE 3D Visualization. **(a)** CARS **(b)** SPA SHAP value contributions of different ginseng seed types under different feature selection methods. **(c)** CARS **(d)** SPA.

**Figure 13a** and **Figure 13b** presents the comparative t-SNE 3D visualizations of the SPA and CARS methods. In the three-dimensional visualization results, the CARS method demonstrated superior intra-class compactness and inter-class separability compared with SPA. Notably, KGS consistently exhibited a stable and compact clustering structure, reflecting stronger robustness and interpretability. In contrast, under the SPA method, the clustering boundaries of MCGS and CGS appeared blurred, while the feature points of AGS were highly scattered, indicating insufficient discriminative power. Overall, the CARS method showed greater advantages in clustering rationality and stability, thereby providing a more reliable basis for algorithm performance comparison and result interpretation.

The characteristic wavelengths selected by the SPA algorithm exhibit strong spectrochemical relevance, as illustrated in **Figure 13c**. The selected bands in the visible region (420 nm, 476 nm, and 580 nm) primarily correspond to seed coat pigments, including chlorophylls, anthocyanins, and carotenoids. These wavelengths are useful for distinguishing seed types and assessing their maturity. The bands in the near-infrared region (829 nm, 1394 nm, 1677 nm, and 1804 nm) are associated with functional groups of major chemical components such as moisture, polysaccharides, lipids, and proteins. These features reflect the internal nutrient reserves or physiological status of the seeds, making them valuable for classifying seed vigor or developmental stages. The short-wave infrared band at 2396 nm corresponds to a strong absorption region and is closely related to combination vibrations of C=O or N–H bonds in proteins and lipid-based compounds. This wavelength plays a key role in identifying nutritional composition and varietal differences among ginseng seeds.

The characteristic wavelengths selected by the CARS algorithm span important absorption regions from the visible to the near-infrared spectrum, as shown in **Figure 13d**. The visible bands (413, 469, 494, 568, 572, and 668 nm) primarily correspond to the characteristic absorption peaks of natural pigments in the seed coat—such as chlorophylls, carotenoids, anthocyanins, and flavonoids—reflecting differences in seed color, maturity, and physiological state. In contrast, the near-infrared bands (924, 928, 1097, 1349, 1671, 1859, and 1873 nm) are mainly associated with overtone and combination absorptions of functional groups such as O–H and C–H, which are closely related to internal seed components including moisture, lipids, and carbohydrates. These spectral features offer a chemically interpretable basis for seed classification at the molecular level, thereby improving the accuracy and robustness of the classification models.

The characteristic wavelengths selected by both the SPA and CARS methods exhibit high chemical consistency, primarily involving functional groups such as C–H, O–H, and C=C. These groups correspond to the characteristic absorptions of lipids, carbohydrates, moisture, and pigment-related compounds, respectively. This indicates that the extracted spectral features effectively capture key molecular signatures related to organic composition and seed coat pigmentation in ginseng seeds, thereby providing a unified and biologically meaningful spectral basis for seed classification.

#### 3.3.2 RF-based classification model for ginseng seeds using hyperspectral data

All spectral data of ginseng seeds were input into the Random Forest (RF) classification model for analysis. The dataset was split into training and testing sets at a ratio of 7:3, with 260 samples randomly selected from each category for model training and validation. The RF model’s hyperparameters, including n_estimators and max_features, were kept at their default settings. The classification performance of the RF model across different seed categories is presented in **Table 5**, showing the evaluation results based on the original hyperspectral bands.

**Table 5:** Comparison of RF classification model performance using hyperspectral bands.

Selection algorithm	Result			
	A	P	R	F1
None	0.8558	0.8587	0.8558	0.8561
SPA	0.8622	0.8636	0.8622	0.8585
CARS	0.8846	0.8996	0.8846	0.8804

As shown in **Table 5**, the classification model based on spectral features demonstrated overall superior performance, with different band selection methods exerting a significant influence on model accuracy. Without feature selection (None), the model achieved relatively high accuracy on the training set but performed poorly on the test set, indicating limited generalization ability. In contrast, the SPA method improved test accuracy to some extent, although the enhancement was limited. Notably, the CARS method achieved the best results across all evaluation metrics, with the test set performance reaching an accuracy of 88.46%, precision of 89.96%, recall of 88.46%, and F1-score of 88.04%, which were all substantially higher than those of other methods. This finding suggests that CARS effectively selects the key spectral bands most relevant to classification, thereby significantly improving the discriminative power of the model. In summary, spectral features, as an important complement to image information, can achieve a more in-depth characterization of ginseng seed traits through appropriate band selection strategies, ultimately enhancing the stability and generalization capability of the model in complex classification tasks.

### 3.4 Comparative analysis of feature fusion

Based on the above analysis, spectral bands reflect the differences among Panax ginseng seed varieties in their reflection, absorption, and transmission properties of incident light, revealing their distinct electromagnetic response characteristics. In contrast, morphological features provide complementary information regarding the seed surface structure and underlying tissue variations, serving as an important supplement to spectral data. Therefore, fusing these two types of features can help construct a more discriminative classification model. In this study, morphological features selected by RFE and SFM algorithms were combined with the 8 key wavelengths extracted by SPA and the 13 feature bands selected by CARS, respectively. These fused feature sets were then used as inputs to the Panax ginseng seed classification models. The classification performance of the RF model under each fusion strategy is detailed in **Table 6**.

**Table 6:** Comparison results of the RF classification model based on fused features.

Selection algorithm	Result			
	A	P	R	F1
None	0.8750	0.8864	0.8750	0.8734
RFE-CARS	0.9281	0.9278	0.9281	0.9276
RFE-SPA	0.7917	0.7951	0.7917	0.7898
SFM-CARS SFM-SPA	0.9103 0.8173	0.9133 0.8283	0.9103 0.8173	0.9104 0.8169

As shown in **Table 6**, feature selection and fusion strategies play a critical role in improving model performance. When using either the full spectral data or the complete set of morphological features alone, the models exhibited excellent results on the training set but suffered from evident overfitting on the test set, indicating limited generalization capability. In contrast, the RFE–CARS fusion strategy, which eliminates redundant morphological features via RFE and simultaneously selects informative spectral bands through CARS, achieved the best performance on the test set, with the accuracy increasing to 92.81%, substantially outperforming the baseline models. This result demonstrates the strong complementarity between image and spectral features, where their integration enhances feature representation, robustness, and classification stability. Moreover, the advantage of RFE–CARS lies in its ability to mitigate the “curse of dimensionality” by retaining only the most relevant information while reducing redundant inputs, thereby improving computational efficiency. Overall, the proposed multimodal feature fusion strategy provides a more generalizable and practical pathway for addressing the challenges of complex crop seed classification.

### 3.5 ORBMO-RF-based classification method for panax ginseng seeds

#### 3.5.1 Comparative experimental analysis of swarm intelligence algorithms

However, key hyperparameters such as n_estimators and max_features often require manual tuning to achieve the best configuration. This manual process is not only inefficient but also prone to subjective bias. To enhance model performance and automate parameter optimization, this study employs swarm intelligence optimization algorithms to tune the RF hyperparameters. During model training and testing, the optimal fused features are still divided into training and testing sets at a 7:3 ratio, where the training set is further evaluated by five-fold cross-validation and the test set is kept fixed and used only for the final performance assessment. To evaluate the effectiveness of different optimization algorithms, three mainstream swarm intelligence algorithms were selected for comparison: RBMO, Marine Predators Algorithm (Marine Predators Algorithm, MPA), and Particle Swarm Optimization (Particle Swarm Optimization, PSO). Each algorithm was applied to classify the fused feature set, with n_estimators fixed at 100 and max_features set to “sqrt”. The comparative results of the models are presented in **Table 7**.

**Table 7:** Comparative results of classification models using optimal feature combinations.

Model	Result			
	A	P	R	F1
RBMO-RF	0.9469	0.9482	0.9469	0.9466
MPA-RF	0.9344	0.9376	0.9344	0.9348
PSO-RF	0.9313	0.9337	0.9313	0.9310
RF	0.9156	0.9153	0.9156	0.9154

As shown in **Table 7**, the RBMO–RF model outperformed the other three models, achieving the highest classification accuracy of 94.7% on the test set, with precision, recall, and F1-score also maintained at consistently high levels. Compared with MPA–RF and PSO–RF, RBMO–RF demonstrated advantages across all evaluation metrics, reflecting superior generalization capability. Furthermore, relative to the baseline RF model without optimization, RBMO–RF achieved a substantial improvement in classification performance. These findings strongly validate the effectiveness and feasibility of the RBMO-based adaptive hyperparameter optimization strategy in enhancing the classification capability of the random forest model.

#### 3.5.2 Comparative analysis of different optimization strategies for feature fusion

To further enhance the global search capability, this study incorporates three mechanisms into the RBMO algorithm: Circle chaos mapping, GSS and ASA perturbation mechanism, forming the improved model ORBMO-RF. Experiments were conducted with each mechanism individually and in pairwise combinations to compare classification accuracies under different strategy configurations. The optimal combination scheme and its corresponding optimization performance are detailed in **Table 8**.

**Table 8:** Comparison of optimization results for different strategy combinations.

Strategy			Evaluation metrics			
Circle chaos mapping	Golden sine search	Adaptive simulated annealing	A	P	R	F1
√			0.9500	0.9514	0.9500	0.9501
	√		0.9437	0.9444	0.9437	0.9436
		√	0.9406	0.9414	0.9406	0.9409
√	√		0.9531	0.9539	0.9531	0.9531
√		√	0.9500	0.9505	0.9500	0.9501
	√	√	0.9594	0.9598	0.9594	0.9592
√	√	√	0.9750	0.9757	0.9750	0.9750

According to the results presented in **Table 8**, the combination of the three strategies (ORBMO-RF) exhibited the best overall performance among all tested approaches. On the test set, its A, P, R, and F1-score reached 97.50%, 97.57%, 97.50%, and 97.50%, respectively, which were markedly superior to those of the original RBMO-RF model and other strategy combinations. These findings indicate that the rational integration of Circle chaotic mapping, GSS and ASA perturbation effectively enhances the global search capability and optimization efficiency of the RBMO algorithm, thereby improving the classification performance of the random forest. Notably, high accuracies were also achieved in the “GSS + ASA” and “Circle + GSS” combinations, further confirming the effectiveness of multi-strategy collaborative optimization in strengthening model generalization and robustness. Therefore, ORBMO-RF can be regarded as a stable and high-performance optimization scheme for ginseng seed classification tasks.

**Figure 14:**
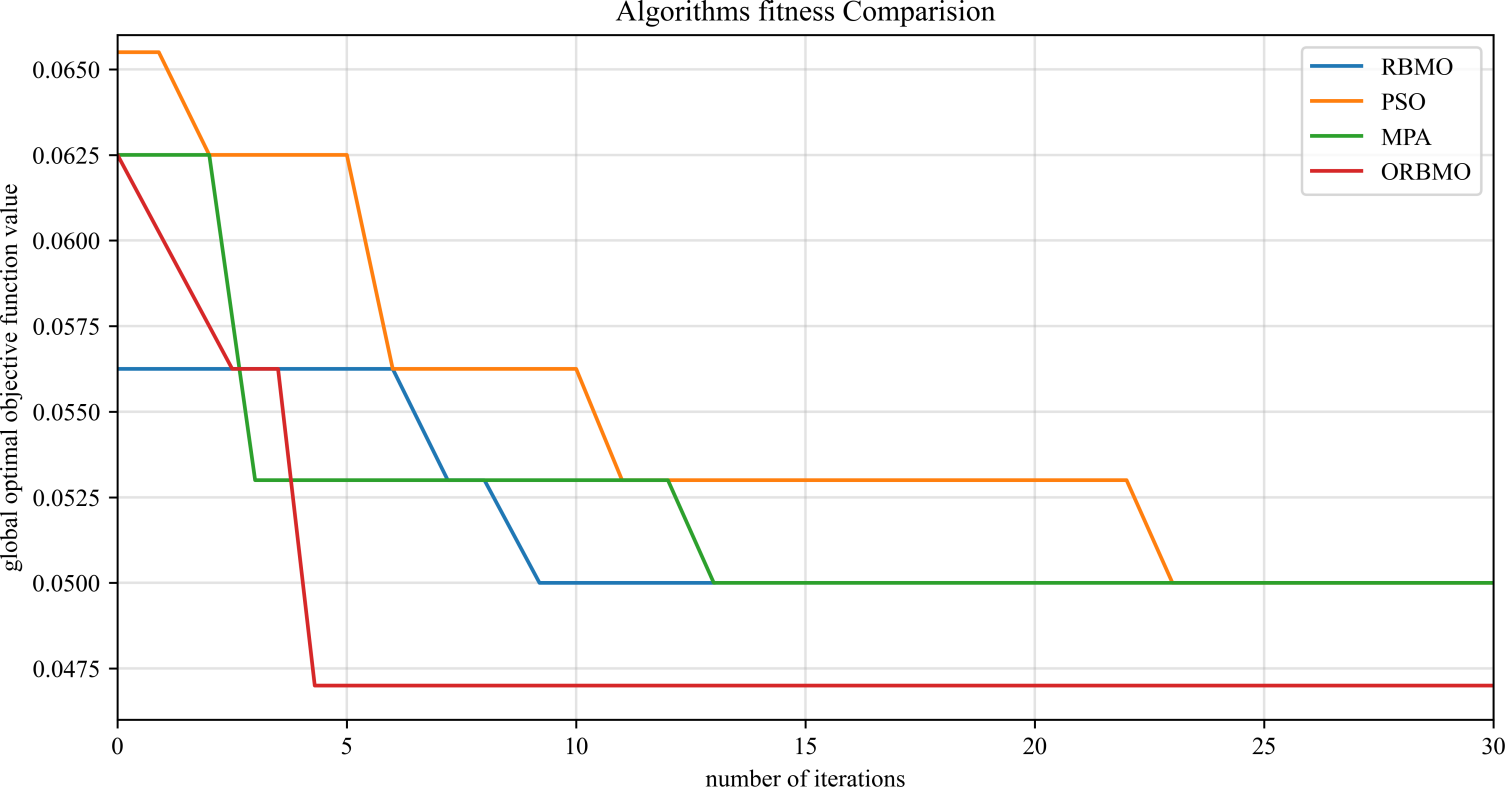
Algorithm fitness curves.

**Figure 14** compares the optimal fitness values achieved by each algorithm, evaluating the convergence behavior and fitness performance of four algorithms. The fitness metric in **Figure 14** is based on the error rate, where lower values indicate better predictive performance. It is evident that the RBMO, PSO, and MPA algorithms tend to converge prematurely to local optima, highlighting the superior global search capability of the ORBMO optimization.

By comparing the classification performance of models with all-feature combinations in **Table 9**, it was observed that ORBMO-RF achieved an A, P, R, and F1-score of 94.06%, 94.19%, 94.06%, and 94.08%, respectively, on the test set. Overall, its performance surpassed that of RBMO-RF, MPA-RF, PSO-RF, and the conventional RF model. This result demonstrates that the feature set derived after feature selection not only reduces redundant information and noise interference but also enhances the model’s ability to exploit key discriminative information, thereby significantly improving classification performance. Compared with models using all features, the multimodal feature set filtered by RFE and CARS achieved substantial dimensionality reduction while maintaining strong biological interpretability, enabling the classification model to capture patterns closely associated with seed quality and chemical composition within a more compact data representation. Therefore, the superior performance of ORBMO-RF can be attributed not only to the efficient hyperparameter optimization achieved by the algorithm but also to the optimized input space enabled by feature selection. These findings highlight the synergistic value of multisource feature integration and intelligent optimization in nondestructive seed classification, providing new insights for developing efficient, stable, and biologically meaningful intelligent recognition models.

**Table 9:** Comparison of classification results using the full feature set.

Model	Result			
	A	P	R	F1
ORBMO-RF	0.9406	0.9419	0.9406	0.9408
RBMO-RF	0.9313	0.9353	0.9313	0.9315
MPA-RF	0.9000	0.9029	0.9000	0.8993
PSO-RF	0.9250	0.9331	0.9250	0.9246
RF	0.8750	0.8864	0.8750	0.8734

#### 3.5.3 Comparative analysis of ORBMO and other hyperparameter optimization strategies

To further examine whether the performance improvement of ORBMO results from effective optimization rather than increased algorithmic complexity, its convergence behavior was compared with several RBMO variants and widely used hyperparameter optimization methods, including IRBMO, MRBMO, Random Search, and Bayesian Optimization. All optimization strategies were evaluated under identical search spaces and computational budgets to ensure comparability.

**Figure 15:**
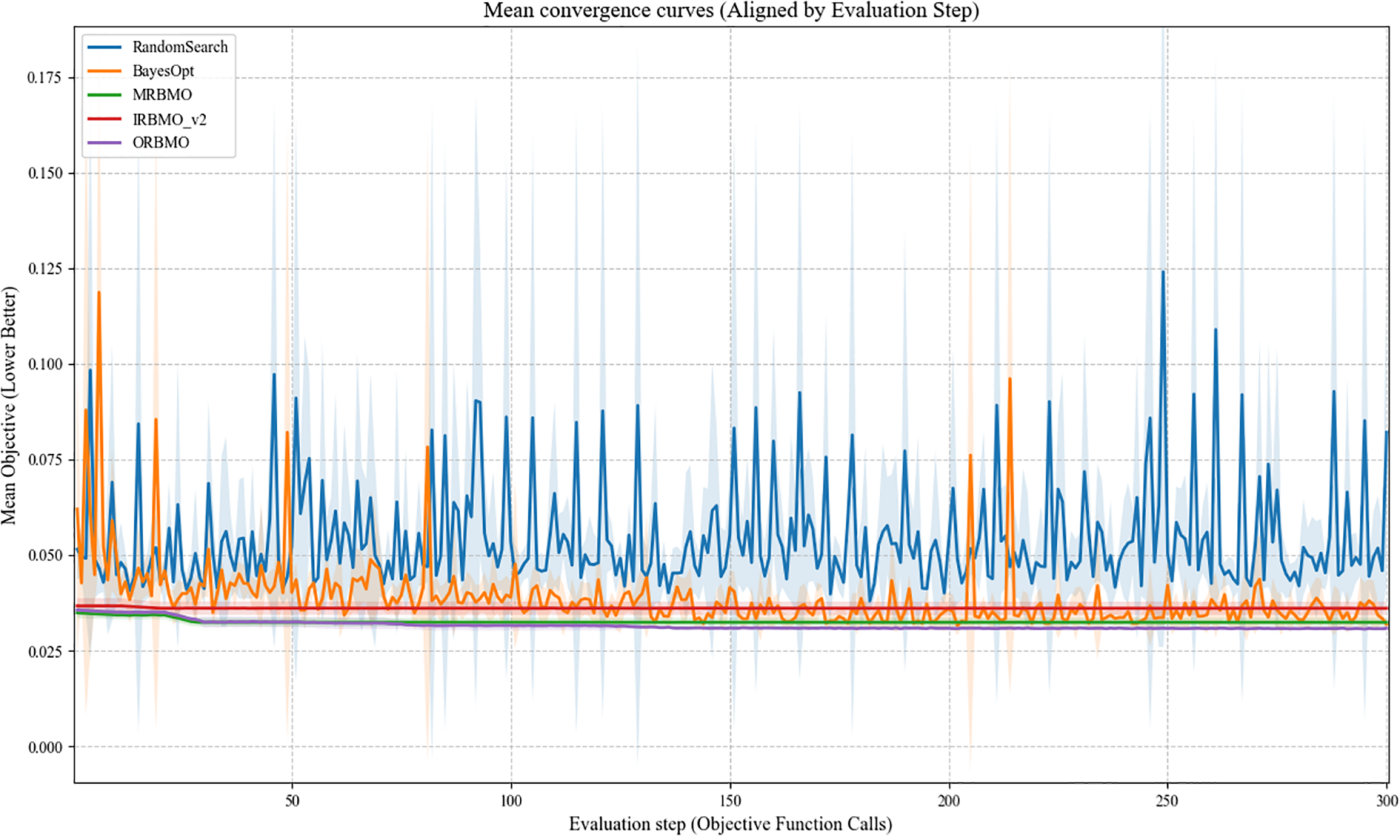
Algorithm average convergence curve.

**Figure 15** presents the convergence curves of different optimization methods aligned by the number of objective function evaluations. For each method, multiple independent runs were conducted, and the mean fitness value was recorded. The fitness metric was defined based on the classification error, where lower values indicate better optimization outcomes. As observed, Random Search shows a slow and highly variable convergence trend, reflecting its stochastic nature. Bayesian Optimization demonstrates relatively stable behavior in the early stages but exhibits limited improvement as the evaluation budget increases.

Compared with standard RBMO and its variants, MRBMO and IRBMO are able to accelerate convergence to some extent; however, their optimization trajectories display noticeable fluctuations across runs, suggesting sensitivity to initialization and local search behavior. In contrast, ORBMO shows a more consistent convergence trend and achieves lower mean fitness values in the later stages of optimization. This indicates that the integration of chaotic initialization, spiral search, and adaptive perturbation contributes to a more stable exploration–exploitation balance during the optimization process.

The convergence curves indicate that ORBMO maintains lower fitness values with reduced variability across runs compared with other optimization strategies under identical evaluation budgets, suggesting a more stable optimization process for the random forest model used in this study.

#### 3.5.4 Comparative analysis of ORBMO-RF and deep learning models

**Table 10** summarizes the results of the five models trained on the same feature set, which combined compressed morphological variables with hyperspectral information. Because the morphological data were reduced to match the spectral sample size, all models worked under the same limited-data conditions. In this setting, ORBMO-RF reached the highest values among the four evaluation metrics, with accuracy, precision, recall, and F1-score all around 0.975, showing a clear improvement over the RF-baseline. The deep learning models—1D-CNN, ResNet1D, and Transformer—produced relatively close results to one another, with accuracies for ResNet1D and Transformer around 0.943, while the 1D-CNN was slightly lower. Although these networks can model complex relationships, their performance tended to level off with the reduced dataset. ORBMO-RF, on the other hand, made more efficient use of the available feature space, which may explain its higher stability in this task. Considering that large annotated seed datasets are often difficult to obtain, these outcomes suggest that a machine-learning framework supported by an adaptive parameter search can be a practical option when both morphological and spectral features are available but sample size remains limited.

**Table 10:** Performance comparison of ORBMO-RF and deep learning models under the same feature set.

Model	Result			
	A	P	R	F1
1D-CNN	0.8394	0.8719	0.8394	0.8363
RF-baseline	0.9156	0.9153	0.9156	0.9154
ResNet1D	0.9432	0.9439	0.9432	0.9431
Transformer-RF	0.9432	0.9450	0.9432	0.9434
ORBMO-RF	0.9750	0.9757	0.9750	0.9750

#### 3.5.5 Classification results analysis

**Table 11** presents a comparison of classification results based on different feature combinations. It is evident that the single morphological feature model (IMG-RBMO-RF) performed poorly, with an accuracy of only 0.6531, indicating that single-source image information is insufficient to support high-precision identification. In contrast, the hyperspectral feature model (Hyperspectral-RBMO-RF) achieved higher classification performance, with an accuracy of 0.9250, suggesting that spectral features can effectively capture variations in seed chemical composition. Notably, when morphological and spectral features were integrated, model performance was further improved, with the Fusion-RBMO-RF model reaching an accuracy of 0.9469. This result confirms the advantage of multisource data fusion in capturing subtle differences among ginseng seeds. Such complementarity arises because morphological features reflect external structural differences, whereas spectral features reveal internal compositional variations, and their combination provides a more comprehensive basis for discrimination. Building on this, the model incorporating the ORBMO algorithm (Fusion-ORBMO-RF) further enhanced classification performance, achieving A, P, R, and F1-score values of 97.50%, 97.57%, 97.50%, and 97.50%, respectively, on the test set, which were significantly superior to those of other comparative models. These findings demonstrate that the synergy between intelligent optimization algorithms and multimodal features can effectively enhance model stability and generalization, offering a feasible and robust technical pathway for efficient and accurate classification of ginseng seeds.

**Table 11:** Comparison of classification results using different feature sets.

Models with different features	Result			
	A	P	R	F1
IMG-RBMO-RF	0.6531	0.6451	0.6531	0.6448
Hyperspectral-RBMO-RF	0.9250	0.9267	0.9250	0.9250
Fusion-RBMO-RF	0.9469	0.9482	0.9469	0.9466
Fusion-ORBMO-RF	0.9750	0.9757	0.9750	0.9750

**Figure 16:**
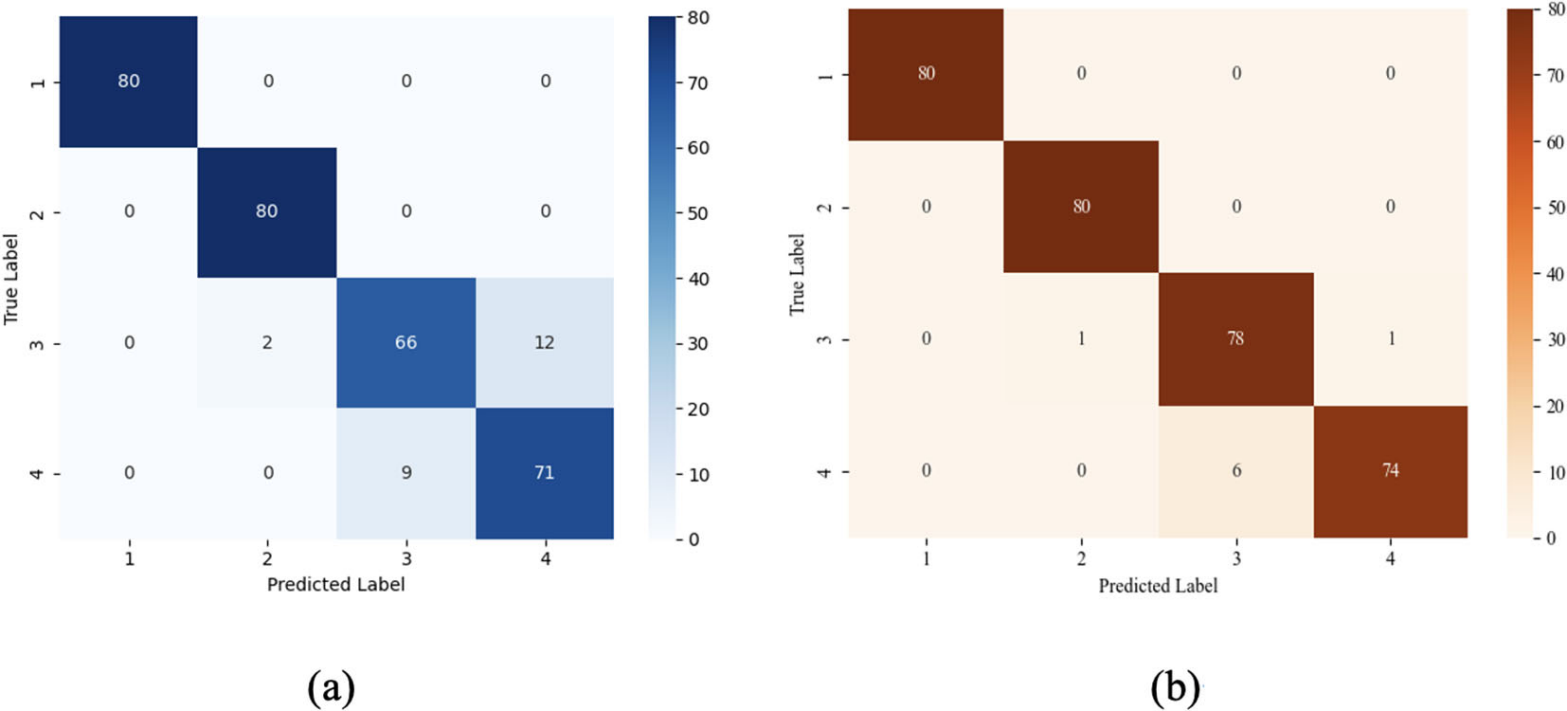
**(a)** Confusion matrix of the RF model on the test set. (1 for KGS, 2 for MCGS,3 for CGS, and 4 for AGS.) **(b)** Confusion matrix of the ORBMO-RF model on the test set. (1 for KGS, 2 for MCGS,3 for CGS, and 4 for AGS.).

The confusion matrix is a widely used evaluation tool in machine learning, designed to assess the performance of classification models across different categories. In this matrix, each column represents the predicted class, while each row corresponds to the actual class. To provide a more intuitive evaluation of model performance, this study compares the confusion matrices of the standard RF model and the proposed ORBMO-RF model, as shown in **Figures 16**. These matrices illustrate the classification performance for different ginseng seed types. Although some misclassifications occur due to the high morphological similarity among certain seed varieties, both models demonstrate strong overall classification accuracy. **Table 12** presents a comparison of different ginseng seed varieties across two models, demonstrating that the ORBMO-RF model exhibits superior recognition capabilities compared to the RF model in distinguishing highly similar seed samples, indicating its strong application potential and promising development prospects in non-destructive seed identification tasks.

**Table 12:** Comparison of classification results for different types of ginseng seed varieties.

Categories	P		R		F1	
	RF	ORBMO-RF	RF	ORBMO-RF	RF	ORBMO-RF
CGS	1.0000	1.0000	1.0000	1.0000	1.0000	1.0000
KGS	0.9756	0.9877	1.0000	1.0000	0.9877	0.9938
MCGS	0.8800	0.9286	0.8250	0.9750	0.8516	0.9512
AGS	0.8554	0.9867	0.8875	0.9250	0.8712	0.9548

As shown in **Table 12**, the ORBMO-RF model outperformed the RF model across all ginseng seed categories. For KGS, both models achieved 100% classification accuracy. However, for CGS, MCGS and AGS, ORBMO-RF yielded higher precision, recall, and F1-scores, with particularly notable improvements observed in MCGS and AGS. Specifically, the F1-scores increased from 85.16% to 95.12% and from 87.12% to 95.48%, respectively. These results indicate that ORBMO can effectively enhance the performance of random forest in classifying difficult categories, further validating the advantages and applicability of this approach for complex cultivar recognition tasks.

## 4 Discussion

This study aimed to ensure the varietal purity of ginseng seeds by addressing the challenge of nondestructive and precise classification among different ginseng varieties. An improved Random Forest (RF) model based on the fusion of morphological and spectral features was proposed. Specifically, morphological and hyperspectral information were integrated to construct a comprehensive feature set, and an optimized Red-Billed Blue Magpie Optimization algorithm (ORBMO) was introduced to intelligently tune the key hyperparameters of the RF model. By incorporating the Circle chaotic map, Golden Sine search strategy, and Adaptive Simulated Annealing perturbation mechanism, ORBMO effectively enhanced the global search capability and convergence stability, thereby reducing the randomness and subjectivity of manual parameter tuning. Through modeling and optimization in a multi-source feature space, the study achieved high-precision automatic classification of ginseng seeds.

Experimental results demonstrated that the ORBMO-RF model integrating morphological and spectral features achieved a classification accuracy of 97.50% on the test set, outperforming models based on single-feature inputs. This confirms the effectiveness of multimodal feature fusion in overcoming traditional classification bottlenecks and improving model discriminative power. The complementary nature of different feature dimensions enabled the model to more accurately capture spectral responses and microstructural differences among Panax ginseng varieties, including Cultivated Ginseng Seeds(CGS), Korean Ginseng Seeds(KGS), Mountain Cultivated Ginseng Seed(MCGS), and American Ginseng Seeds(AGS) thus providing new insights into distinguishing morphologically similar but chemically diverse seed varieties.

The significance and value of this research lie in two main aspects. Methodologically, it is the first to apply an improved swarm intelligence optimization algorithm (ORBMO) to ginseng seed classification, establishing a high-precision multimodal fusion framework that can be generalized to other agricultural intelligence and parameter optimization tasks. Practically, the proposed approach ensures the varietal purity of ginseng seeds, preventing problems such as reduced germination rate, uneven plant growth, and variability in medicinal components caused by seed mixture. This contributes to improved breeding efficiency, standardized cultivation, and enhanced consistency in pharmacological efficacy.

Despite achieving high accuracy, the study has certain limitations. The current feature set can be further expanded to improve model robustness and generalization in distinguishing overlapping or minority classes. Moreover, the experiments were primarily conducted under laboratory conditions, lacking validation in complex production environments and without fully accounting for the effects of storage, aging, frost damage, or fungal infection on feature expression and classification performance. Future research will focus on constructing a multi-factor integrated classification framework that combines morphological, spectral, environmental, and physiological variables to enable dynamic identification under varying conditions. Incorporating fine-grained phenotypic and environmental parameters is expected to enhance the model’s practicality and interpretability, providing a solid foundation for precise germplasm identification and intelligent breeding of medicinal plants.

## Data Availability

The raw data supporting the conclusions of this article will be made available by the authors, without undue reservation.
