# Supplementary material for: ORBMO-RF: a non-destructive classification method for ginseng seeds based on multimodal fusion and improved red-billed blue magpie optimization algorithm
Source: Front Plant Sci. 2026 Jan 14;16:1743311. doi: 10.3389/fpls.2025.1743311 (PMC12847255; doi:10.3389/fpls.2025.1743311)
Supplement: Supplementary file 1 [file Table1.docx]

**Supplementary Materials**

Table 1 Definitions and Mathematical Expressions of Geometric and Texture Morphological Features

|  |  | Feature Name (Abbreviation) | Meaning | Formula for Calculation |
| --- | --- | --- | --- | --- |
| Morphological Characteristics | Geometric characteristics | Circumference (S) | When calculating the circumference of ginseng seeds, coordinate points extracted from the seed edges are used to generate a continuous curve through interpolation. The final circumference value is obtained by approximating the length of this curve. | - |
|  |  | Area (A) | By counting the pixels within the seed outline area and summing their areas, the surface area of ginseng seeds can be efficiently calculated. | - |
|  |  | Long axis length (L) | By performing elliptical fitting on the ginseng seed profile, the angle between the major axis and the horizontal direction is obtained. Using a rotational profile method, a rectangle parallel to the seed profile is derived. Calculating the Euclidean distance to the center point of the longer side yields the length of the major axis. | - |
|  |  | Short Shaft Length (W) | The short axis length of the seed is obtained by calculating the Euclidean distance to the center point of the short side of the bounding rectangle parallel to the primary direction of the seed's contour. | - |
|  |  | Inscribed circle radius (r) | The radius of the inscribed circle refers to the radius of a circle that touches all sides of a polygon, and it can describe the compactness of the polygon's shape. | $r=\frac{2\times A}{S}$ |
|  |  | Aspect Ratio (K) | The aspect ratio denotes the ratio of the major axis length to the minor axis length of the seed outline area, used to describe the seed's contour shape. | $K=\frac{L}{W}$ |
|  |  | Dispersion (e) | Dispersion, also known as complexity, primarily expresses the size of the perimeter of a given area. | $e=\frac{S^{2}}{A}$ |
|  |  | Roundness (C) | Represents the degree of proximity between the ginseng seed region and the circular area, thereby reflecting the complexity of the ginseng seed region. | $C=\frac{4\times\pi\times A}{S^{2}}$ |
|  |  | Elongation (E) | The elongation ratio, also known as the eccentricity, is a key parameter describing the morphological characteristics of ginseng seeds, determined by the ratio of the seed's short axis to its long axis. | $E=\frac{W}{L}$ |
|  |  | Rectangularity (R) | Rectangularity is typically used to measure how closely an object approximates a rectangle, reflecting the compactness and regularity of its outline. It indicates the degree of curvature and complexity in the object's contour. | $R=\frac{A}{L\times W}$ |
|  |  | Equivalent Circle Diameter (Ed) | The diameter of a circle with the same area as the ginseng seed region | $Ed=\sqrt{\frac{4\times A}{\pi}}$ |
|  |  | Conservative Moment (Hu) | Invariant moments, also known as Hu moments, are a feature description method used in image processing and pattern recognition. They are constructed using second- and third-order central moments to form seven invariant moments. | - |
|  | Texture characteristics | Contrast | Contrast reflects the sharpness and depth of texture in an image. A higher contrast value indicates more pronounced surface texture variations in the ginseng seed image. | $Contrast=\sum_{i,j=0}^{N-1} P(i,j)\times{(i-j)}^{2}$ |
|  |  | Dissimilarity | Dissimilarity serves as a metric for describing the degree of difference between image texture features. In the grayscale co-occurrence matrix, elements farther from the diagonal represent differences between distinct grayscale levels, which can be quantified using dissimilarity. | $Dissimilarity=\sum_{i,j=0}^{N-1} P(i,j)\times\left\vert i-j \right\vert$ |
|  |  | Homogeneity | Quantifies the similarity of gray-scale values among neighboring pixels in an image, reflecting the local uniformity and smoothness of texture. | $Homogeneity=\sum_{i,j=0}^{N-1} \frac{P(i,j)}{1+{(i-j)}^{2}}$ |
|  |  | ASM | The second-order moment describes the local contrast between gray-level pairs in an image, focusing on capturing local details. | $ASM=\sum_{i,j=0}^{N-1} {(P(i,j))}^{2}$ |
|  |  | Energy | Energy is used to measure the frequency distribution of gray-level pairs in an image, primarily describing the overall texture distribution. | $Energe=\sqrt{ASM}$ |
|  |  | Correlation | The correlation reflects the degree of similarity among elements in the grayscale co-occurrence matrix across different directions. The magnitude of the correlation value indicates the local grayscale correlation within the image. | $\begin{aligned} Correlation=\sum_{i,j=0}^{N-1} P\left( i,j \right)\times\left[ \frac{\left( i-\mu_{i} \right)\times\left( j-\mu_{j} \right)}{\sqrt{\delta_{i}^{2}}\times\delta_{j}^{2}} \right] \\ \mu_{i}=\sum_{i,j=0}^{N-1} i\times P\left( i,j \right)\# \\ \mu_{j}=\sum_{i,j=0}^{N-1} j\times P\left( i,j \right) & \\ \delta_{i}^{2}=\sum_{i,j=0}^{N-1} P(i,j)\times\left( i-\mu_{i} \right)^{2}& \\ \delta_{j}^{2}=\sum_{i,j=0}^{N-1} P(i,j)\times\left( j-\mu_{j} \right)^{2} \end{aligned}$ |
|  |  | LBP | Local Binary pattern is a method that reveals local texture features by comparing the grayscale values of specific pixels with their surrounding pixels. When calculating LBP features, an image is typically divided into a number of regions. | - |
